# Whole‐genome sequencing reveals small genomic regions of introgression in an introduced crater lake population of threespine stickleback

**DOI:** 10.1002/ece3.2047

**Published:** 2016-03-02

**Authors:** Kohta Yoshida, Ryutaro Miyagi, Seiichi Mori, Aya Takahashi, Takashi Makino, Atsushi Toyoda, Asao Fujiyama, Jun Kitano

**Affiliations:** ^1^ Division of Ecological GeneticsDepartment of Population GeneticsNational Institute of GeneticsMishimaShizuokaJapan; ^2^ Evolutionary Genetics LaboratoryDepartment of Biological SciencesTokyo Metropolitan UniversityHachiojiTokyoJapan; ^3^ Biological LaboratoryGifu‐keizai UniversityOgakiGifuJapan; ^4^ Division of Ecology and Evolutionary BiologyGraduate School of Life SciencesTohoku UniversitySendaiMiyagiJapan; ^5^ Comparative Genomics LaboratoryCenter for Information BiologyNational Institute of GeneticsMishimaShizuokaJapan; ^6^ Department of GeneticsSOKENDAI (The Graduate University for Advanced Studies)MishimaShizuokaJapan

**Keywords:** Biological invasion, conservation genomics, exotic species, fisheries, hybridization, Japan Sea stickleback, next‐generation sequencer, rapid evolution

## Abstract

Invasive species pose a major threat to biological diversity. Although introduced populations often experience population bottlenecks, some invasive species are thought to be originated from hybridization between multiple populations or species, which can contribute to the maintenance of high genetic diversity. Recent advances in genome sequencing enable us to trace the evolutionary history of invasive species even at whole‐genome level and may help to identify the history of past hybridization that may be overlooked by traditional marker‐based analysis. Here, we conducted whole‐genome sequencing of eight threespine stickleback (*Gasterosteus aculeatus*) individuals, four from a recently introduced crater lake population and four of the putative source population. We found that both populations have several small genomic regions with high genetic diversity, which resulted from introgression from a closely related species (*Gasterosteus nipponicus*). The sizes of the regions were too small to be detected with traditional marker‐based analysis or even some reduced‐representation sequencing methods. Further amplicon sequencing revealed linkage disequilibrium around an introgression site, which suggests the possibility of selective sweep at the introgression site. Thus, interspecies introgression might predate introduction and increase genetic variation in the source population. Whole‐genome sequencing of even a small number of individuals can therefore provide higher resolution inference of history of introduced populations.

## Introduction

An increasing number of organisms have been anthropogenically transferred from native habitats to novel environments. Such introduced species or populations pose a major threat to biological diversity (Sakai et al. 2001; Pejchar and Mooney 2009; Ricciardi et al. 2013). Initial colonization by an introduced population often involves a population bottleneck (Allendorf and Lundquist 2003), which mostly reduces genetic diversity and may also reduce the ability to adapt to diverse environments. However, recent studies have revealed that reduction in genetic variation of introduced populations is not as common as expected. For example, it is reported that only 37% of introduced populations showed lower genetic diversity compared with the source populations (Roman and Darling 2007).

Hybridization between divergent lineages and/or multiple introductions often underlie the maintenance of relatively high genetic variance and adaptation in invasive species (Lee 2002; Kolbe et al. 2004; Roman and Darling 2007; Dlugosch and Parker 2008; Forsman 2014; Rius and Darling 2014; Bock et al. 2015). Hybridization between distantly related populations not only increases standing genetic variation but also sometimes leads to the formation of hybrid populations with extreme (transgressive) phenotypes (Rieseberg et al. 1999; Seehausen 2004; Nolte et al. 2009). The history of hybridization is generally inferred with population genetic methods. During the last few decades, genetic analysis of introduced populations has been conducted mainly using molecular markers, such as microsatellites and amplified fragment length polymorphisms. Although such markers are useful for obtaining basic information about population genetics parameters, they have limitations (Harrisson et al. 2014). First, a small number of markers represents only a tiny portion of the genome and may fail to identify genomewide diversity or phenotypic variation (Reed and Frankham 2001). Second, these markers themselves are assumed to be neutral, so they cannot provide direct information about genetic variations that are important for adaptation (Ouborg et al. 2010; Angeloni et al. 2012). Therefore, a few randomly chosen markers may not be a good predictor of adaptive potential.

Massively parallel or next‐generation sequencing technologies have recently emerged and now make it possible to conduct population genetic analysis at the whole‐genome sequence level (Angeloni et al. 2012; Ekblom and Wolf 2014). An increased number of nucleotides that can be analyzed will not only increase the accuracy of the estimation of historical demography but also help to identify loci that are important for adaptation (Primmer 2009; Allendorf et al. 2010; Ouborg et al. 2010; Ekblom and Wolf 2014; Harrisson et al. 2014; Bock et al. 2015). However, the cost of whole‐genome sequencing of a large number of individuals with sufficient coverage depth for population genetic analyses is often prohibitive (Davey et al. 2011). Therefore, there is debate about the appropriate use of genomic technologies. One solution is to use methods intermediate between whole‐genome sequencing and traditional marker‐based analysis. Reduced‐representation sequencing, such as restriction site‐associated DNA (RAD) sequencing (Baird et al. 2008) and double digest RAD (ddRAD) sequencing (Peterson et al. 2012), are now commonly used. However, reduced‐representation sequencing targets only limited regions, for example, around restriction sites conserved among the individuals analyzed (Davey et al. 2011; Gautier et al. 2013) and possibly overlooks small genomic regions important for adaptation. Therefore, it is important to know whether whole‐genome sequencing of even a small number of individuals can provide insights that could not be obtained from traditional marker‐based genetic analysis or even reduced‐representation sequencing of many individuals.

Threespine sticklebacks are well‐researched organisms in ecology and evolutionary biology (Bell and Foster 1994; Schluter 2000; McKinnon and Rundle 2002). Anadromous or marine forms of *G. aculeatus* are widely distributed in northern Pacific and Atlantic coasts. Colonization by these ancestral forms has led to the evolution of derived freshwater forms in a variety of freshwater ecosystems. Recent genetic studies have revealed that standing genetic variation in source marine populations contributes to parallel adaptation to freshwater environments (Colosimo et al. 2005; Miller et al. 2007; Kitano et al. 2010; Jones et al. 2012a, 2012b; Roesti et al. 2014; Terekhanova et al. 2014), although de novo mutations can also underlie repeated phenotypic evolution (Chan et al. 2010). In addition to native freshwater populations, introduced freshwater lake populations have been reported (Bell and Aguirre 2013). In the case of one small Alaskan lake population, rapid evolution of armor plates has been observed within 12 years after introduction, which is thought to be caused by selection on the standing genetic variation of a major gene controlling armor plate morphology (Bell et al. 2004; Le Rouzic et al. 2011). In another case in Switzerland, hybridization between two distinct lineages might play an important role in the establishment of an introduced lake population (Lucek et al. 2010). These data are consistent with the idea that standing genetic variation is important for rapid adaptation to novel environments (Barrett and Schluter 2008).

**Figure 1:**
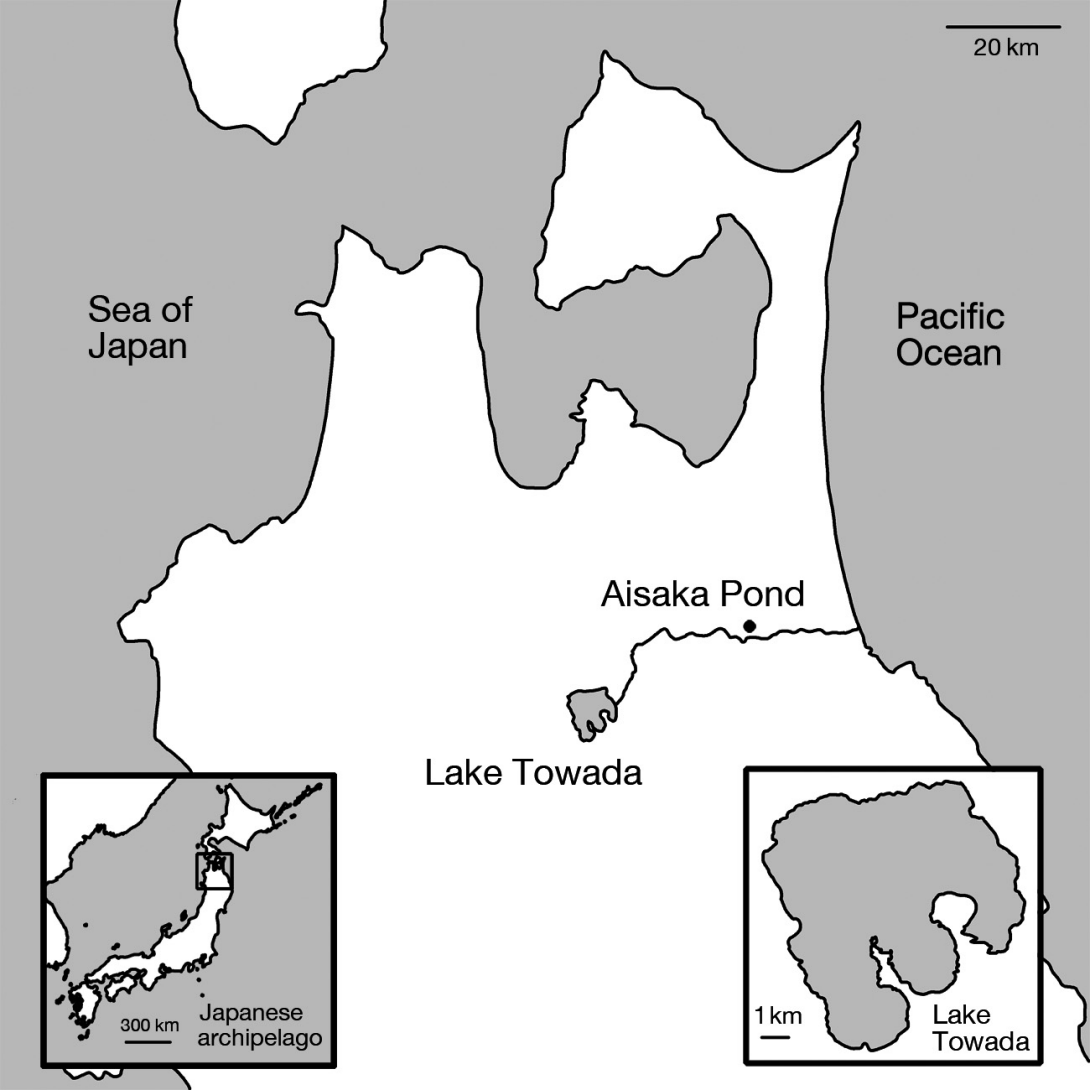
Map showing the location of Lake Towada and Aisaka Pond in Mainland Japan.

Introduced threespine stickleback populations occur in several crater lakes across the Japanese archipelago (Adachi et al. 2012). Previously, we conducted genetic analysis of a crater lake population (Lake Towada) with nine microsatellite markers on 15 individuals (Adachi et al. 2012; Cassidy et al. 2013). Our genetic analysis showed that this population has low genetic diversity; for example, comparison with other Japanese populations showed that the allelic richness of the Lake Towada population was much less than that of the anadromous forms and was the lowest among freshwater populations examined except for one endangered population (Cassidy et al. 2013). Furthermore, the Towada population showed genetic similarity with a nearby population occurring on a trout farm in Aisaka, suggesting that this is the source population (Fig. 1; Adachi et al. 2012). These genetic data are consistent with historical literature documenting that trout were introduced into Lake Towada for aquaculture multiple times (Hikita and Taniguchi 1959; Tokui 1959, 1964). Within 20 years of introduction, morphological and ecological changes have been observed in the Lake Towada population (Adachi et al. 2012). In contrast to the small pond in Aisaka, Lake Towada has a maximum depth of 326.8 m and a surface area of 59.8 km^2^ (Larson 1989). Stickleback in Lake Towada population shifted from benthivory to planktivory and have undergone changes in several morphological traits involved in trophic ecology. Rates of infection by the *Schistocephalus* parasite have also varied during the last three decades (Adachi et al. 2012). How a population introduced from such a small and shallow pond in the trout farm rapidly adapted to the much larger and deeper crater lake and became established remains elusive.

In this study, we first conducted whole‐genome sequencing of four individuals from Lake Towada and four individuals from the trout farm source (Aisaka Pond) population. We found that some genomic regions, which showed high genetic diversity in contrast to the rest of the genome in the Lake Towada and the Aisaka Pond populations, resulted from recent introgression from a different species, the Japan Sea stickleback (*Gasterosteus nipponicus*) (Higuchi and Goto 1996; Kitano et al. 2007, 2009; Higuchi et al. 2014). In contrast to *G. aculeatus*, no freshwater populations of the Japan Sea stickleback lineage have been thus far reported (Higuchi and Goto 1996; Cassidy et al. 2013; Ravinet et al. 2014). *Gasterosteus nipponicus* is thought to have diverged from *G. aculeatus* a few million years ago (Kitano et al. 2009). When they occur in sympatry, these two species are reproductively isolated with low levels of hybridization (Kitano et al. 2009). To investigate whether an introgression site underwent selective sweep, we increased the sample sizes and conducted targeted amplicon (PCR product) sequencing for the regions within and around an introgression site.

## Materials and Methods

### Whole‐genome sequencing

We conducted whole‐genome sequencing of four females from the Lake Towada population and four females from the Aisaka Pond population (Fig. 1; Table 1). Lake Towada stickleback were collected at the western side of the lake near Mount Namari (40.445N, 140.841E) in June 2010 by a local fisherman using gill nets. Aisaka Pond stickleback were collected from a small pond on a trout farm (40.592N, 141.221E) in October 2010 using hand nets (Adachi et al. 2012). Genomic DNA was isolated with a Qiagen DNeasy Blood & Tissue Kit (Qiagen, Valencia, CA). Genomic libraries were constructed with a TruSeq DNA Sample Prep Kit (Illumina, San Diego, CA), and the libraries were run on a HiSeq2000 in 100‐bp paired‐end mode (one fish per one lane) as described previously (Yoshida et al. 2014).

**Table 1:** Coverage and mapping rates of sequence reads, substitution rates compared to the reference stickleback genome, and proportion of heterozygous sites in the sequenced individuals of Lake Towada and Aisaka Pond populations

Population	Individual number	Average coverage	Total Mapped region (%)	Mapped region with 20–200 coverage (%)	Substitution rate (%)	Proportion of heterozygous sites
Lake Towada	1	52	88	84	0.47	0.0014
Lake Towada	2	42	88	83	0.46	0.0016
Lake Towada	3	50	88	83	0.48	0.0018
Lake Towada	4	47	88	84	0.47	0.0014
Aisaka Pond	1	49	88	83	0.48	0.0017
Aisaka Pond	2	48	88	84	0.46	0.0015
Aisaka Pond	3	44	88	83	0.46	0.0016
Aisaka Pond	4	57	88	84	0.47	0.0016

Mapping of sequence reads was conducted as described previously (Yoshida et al. 2014). Briefly, sequence reads with low quality scores (<0.05) and two or more ambiguous nucleotides at the end were trimmed. Trimmed sequence reads were mapped to the stickleback reference genome (ver. BROADS1.56) generated from an Alaskan lake female annotated using a general feature format file (ver. BROADS1.63) in CLC Genomics Workbench Software 6 (CLC bio, Aarhus, Denmark). Reference files were downloaded from ENSEMBL (http://www.ensembl.org). Repeat sequences were masked in the reference sequence. The mapping parameters were as follows: similarity = 0.8, length fraction = 0.5, insertion cost = 3, deletion cost = 3, mismatch cost = 2, color space alignment = no, global alignment = no, override paired distance = no, min distance = 100 bp, and max distance = 1000 bp. To analyze the coverage, we exported the mapping data as bam files and extracted the coverage data at each nucleotide site using BEDTools (Quinlan and Hall 2010). Average coverage of the whole‐genome sequence was calculated with custom Perl scripts. In the subsequent analyses, we used nucleotide sites where all analyzed individuals had 20–200 times coverage.

For SNP analysis of Lake Towada and Aisaka Pond stickleback, SNP calling was conducted using the Probabilistic Variant Detection algorithm implemented in CLC Genomics Workbench. The parameters were as follows: ignore nonspecific matches = yes, ignore broken pairs = yes, minimum coverage = 20, variant probability = 90.0, require presence in both forward and reverse reads = yes, maximum expected variants = 2, and ignore quality scores = no. SNP data was exported as a text file, and subsequent SNP analyses were conducted using custom Perl scripts. Data from a previous study of *G. nipponicus*, anadromous *G. aculeatus,* and *Gasterosteus wheatlandi* (Yoshida et al. 2014) were additionally used for SNP analyses. *G. nipponicus* and *G. aculeatus* correspond to the Japan Sea stickleback and the Pacific Ocean stickleback, respectively, previously reported by Yoshida et al. (2014).

For the sliding window analysis of the heterozygosity within populations (*h*), we calculated average heterozygosity (Nei 1978; Tajima 1989),

$$\hat{h}=\sum_{i=1}^{S}\frac{n(1-\sum_{j}x_{ji}^{2})}{n-1}$$

where *S* is the number of segregation sites, *n* is the chromosome number, and *x*_*ji*_ is the sample frequency of the *j*th allelic nucleotide in the *i*th segregation site. The use of average heterozygosity is recommended as the index of genetic diversity when the sample size is small (Nei 1987). We calculated the average heterozygosity using SNP data from four individuals in each population. In the previous study, we sequenced the genome of five females of anadromous *G. aculeatus* (Yoshida et al. 2014). To use the same number of individuals as the Lake Towada population, we selected four individuals with the highest average coverage for calculation of average heterozygosity (POF1, POF2, POF3, and POF5; see Table 1 in Yoshida et al. 2014). For the sliding window analysis of the proportion of heterozygous sites, we counted heterozygous SNPs and calculated the proportion of heterozygous SNPs at validated nucleotide sites.

To find the introgression sites from *G. nipponicus* in the genome of Lake Towada and Aisaka Pond stickleback, we calculated the proportion of genotypes with *G. nipponicus*‐specific alleles. We focused on introgression sites >10 kb in length. Detection of the introgression sites had four steps. First, we searched for a site where the nucleotide is different between *G. aculeatus* and *G. nipponicus* but is fixed within each species using previously resequenced genomes of 10 individuals of *G. nipponicus* and 10 individuals of anadromous *G. aculeatus* (Yoshida et al. 2014). The sex chromosomes, LG9 and LG19, were excluded from the introgression analysis because X and Y chromosomes can differ even within a species, so it is difficult to find appropriate species‐specific alleles on sex chromosomes. Next, among these sites, we searched for sites where mutation occurred in the *G. nipponicus* lineage rather than the *G. aculeatus* lineage: We searched for sites where the *G. nipponicus* sequence is different from that of the out‐group species *G. wheatlandi* (i.e., a site with a *G. nipponicus*‐specific allele). Third, for each individual from the Lake Towada and Aisaka Pond populations, we determined whether the genotype at each nucleotide site is a homozygote of a *G. aculeatus* allele, a homozygote of a *G. nipponicus* allele, or a heterozygote.

Finally, we conducted a sliding window analysis of the proportion of each genotype across the genome with a window of 500 kb and a step size of 100 kb. Using the 500‐kb sliding window analysis, we screened for regions where more than 2% of the nucleotide sites in a 500‐kb window have *G. nipponicus*‐specific alleles in either a heterozygous or homozygous state as candidate regions containing introgression sites in at least one individual in a population. This 500‐kb sliding window analysis identified 36 candidate genomic regions in the Lake Towada population and 40 candidate regions in the Aisaka Pond population. Then, to narrow down the introgression sites, we conducted a 1‐kb sliding window analysis on these candidate regions and identified genomic regions that have five or more segregating sites with *G. nipponicus*‐specific alleles and are >10 kb in length. The borders of the introgression sites were defined as the loci where a dominant genotype (>50%) of the segregating sites changed from one species to another in the 1‐kb sliding window. In several figures, we used the window of 10 kb and a step size of 10 kb only for visualization (see figure legends).

### in silico RAD and ddRAD sequencing

We conducted in silico RAD (Baird et al. 2008) and ddRAD sequencing (Peterson et al. 2012) to test whether these methods have the potential to detect the *G. nipponicus* introgression sites. The analysis consisted of two steps. We first searched for restriction sites within the introgression sites that we identified in the Towada population. For in silico RAD sequencing, we used SbfI sites, while for the in silico ddRAD sequencing, pairs of EcoRI‐MspI and SphI‐MspI were used because these enzymes are frequently used (Baird et al. 2008; Peterson et al. 2012). We excluded restriction sites that contained one or more fixed nucleotide differences between the two species because only restriction sites conserved between species are informative for RAD or ddRAD sequence analysis (Fig. S1).

The final step of in silico analysis is corresponding to HiSeq2000 paired‐end sequencing (2 × 100 bp) of the virtual RAD and ddRAD libraries. For the in silico RAD sequencing, we counted the number of sites where a nucleotide in *G. nipponicus* is different from that in *G. aculeatus* and *G. wheatlandi,* which we used in the identification of introgression sites, within the regions 100 bp upstream and 100 bp downstream of the restriction sites as well as within the 100‐bp fragments located 400 bp away from the restriction site: The reason why we also used the 100‐bp fragments located 400 bp away from the restriction site is because we simulated 2 × 100 bp paired‐end sequencing here. For the in silco ddRAD sequencing, we computationally selected DNA fragments with a length of 300 ± 36 bp sandwiched between sites recognized by two different restriction enzymes, either EcoRI‐MspI or SphI‐MspI (300 ± 36 bp was used according to the previous simulations conducted in the original paper of ddRAD (Peterson et al. 2012)), and counted the number of *G. nipponicus* diagnostic loci within 100 bp of both ends of the fragment.

### Amplicon sequencing

We conducted targeted amplicon sequencing of a genomic region around a *G. nipponicus* introgression site on LG17 (1–4,000,000 bp). We designed 48 primer pairs that amplified PCR products with a fragment length of 550–850 bp. These primer pairs were chosen within exons, include one or more segregating sites that were identified in the whole‐genome sequence data of the four Lake Towada individuals, and are nearly evenly spaced with an average distance of 87,644 ± 51,447 (SD) bp. Genomic DNA of 50 Lake Towada stickleback was extracted using a Qiagen DNeasy Blood & Tissue Kit and quantified using a Quant‐iT PicoGreen dsDNA Assay Kit (Life Technologies, Carlsbad, CA). Multiplexed PCR was conducted using a Fluidigm Access Array System (Fluidigm, South San Francisco, CA). Before running PCR on the Fluidigm Access Array System, quality controls of genomic DNA and primer pairs were conducted following the manufacturer's instructions (“two‐primer target‐specific PCR amplification” in “Access Array System for Illumina Sequencing Systems”). Briefly, to test the quality of genomic DNA, PCRs were conducted using one primer pair (3035F and 3035R in Table S1) on 50 genomic DNA samples on a Veriti 384 well thermal cycler (Life Technologies) with one cycle of 50°C for 2 min, 70°C for 20 min and 95°C for 10 min; 10 cycles of 95°C for 15 sec, 60°C for 30 sec, and 72°C for 1 min; two cycles of 95°C for 15 sec, 80°C for 30 sec, 60°C for 30 sec, and 72°C for 1 min; eight cycles of 95°C for 15 sec, 60°C for 30 sec, and 72°C for 1 min; two cycles of 95°C for 15 sec, 80°C for 30 sec, 60°C for 30 sec, and 72°C for 1 min; eight cycles of 95°C for 15 sec, 60°C for 30 sec, and 72°C for 1 min; and five cycles at 95°C for 15 sec, 80°C for 30 sec, 60°C for 30 sec, and 72°C for 1 min using the FastStart High Fidelity PCR System, dNTPack (Roche, Mannheim, Germany) and 20× Access Array Loading Reagent (Fluidigm), followed by analysis of the PCR products on an Agilent 2100 Bioanalyzer System with an Agilent DNA 1000 Kit (Agilent Technologies, Santa Clara, CA). We selected 48 DNA samples giving rise to PCR products with concentrations >4 nmol/L. All primer pairs were also validated using PCR under the same reaction conditions shown above with genomic DNA of one randomly chosen individual. Amplification of appropriate DNA fragments was confirmed by direct Sanger sequencing of the PCR products. We tested 57 pairs and selected 48 primer pairs that gave rise to a single PCR product of the expected length.

The parallel PCR amplification of 48 genomic regions from 48 samples (2304 reactions in total) was performed using a Fluidigm Access Array System (Fluidigm) in accordance with the manufacturer's instructions. Briefly, PCR was performed using two target‐specific primers for each sample (Table S1). Amplification reactions were conducted in 35 nL reaction volume on a 48.48 Access Array Integrated Fluidic Circuits (Fluidigm) using the FastStart High Fidelity PCR System, dNTPack (Roche) and 20× Access Array Loading Reagent (Fluidigm) with the same cycle conditions described above. We used 1.3 ng of genomic DNA per reaction. For each DNA sample, pooled amplicons were quantified using an Agilent 2200 TapeStation (Agilent Technologies). PCR products were purified using an AMPure XP Kit (Beckman Coulter, Brea, CA) and quantified with a Quant‐iT PicoGreen dsDNA Assay Kit. An equal amount of purified PCR products (1 ng) was subjected to sequence library construction using a Nextera XT DNA Sample Preparation Kit (Illumina). The Nextera XT Index Kit (Illumina) was used for labeling 48 individuals with different dual barcodes. Paired‐end sequencing (2 × 300 bp) was conducted on a MiSeq sequencer (Illumina) with a MiSeq Reagent Kit v3 (Illumina).

The amplicon sequence reads were mapped to the reference genome sequence of LG17 using the CLC Genomics Workbench 7 with the same parameters used for whole‐genome sequencing. SNP calling was conducted with the basic variant detection tool implemented in CLC Genomics Workbench 7 with the following parameters: ploidy = 2, ignore positions with coverage above = 100,000, restrict calling to target regions = not set, ignore broken pairs = no, ignore nonspecific matches = reads, minimum coverage = 20, minimum count = 5, minimum frequency = 25, base quality filter = no, read direction filter = no, relative read direction filter = no, and remove pyro‐error variants = no. The data of read coverage and variants were exported as text files and used in the subsequent analysis.

### Population genetic analysis using amplicon sequence data

Using the obtained amplicon sequence data from 48 individuals, we first calculated the frequency of *G. nipponicus* alleles within and around the introgression site IS1 (1.169–1.622 Mb) on LG17. SNPs that fulfilled the following three criteria were used for the analysis: (1) coverage depth was >20 in all 48 individuals; (2) the SNP was not included in insertion, deletion, and multiple nucleotide polymorphism (MNP; i.e., mutation of two or three consecutive nucleotides); (3) only two variants exist among all 48 individuals (i.e., bi‐allelic). Called variant types (SNP, MNP, insertion, or deletion) in the variant detection tools often varied among individuals at loci where MNP is present, so an automatic decision of genotypes is difficult there and we excluded SNPs included in MNP. The allele frequency was calculated at the sites with fixed nucleotide difference between the two species. Most amplicons included multiple informative SNPs. A test for Hardy–Weinberg equilibrium was conducted with an exact test implemented in Haploview (Barrett et al. 2005).

For linkage disequilibrium analysis, all SNP data (not limited to the sites with fixed nucleotide differences) were phased with fastPHASE v. 1.4 (Scheet and Stephens 2006) to infer the haplotypes. SNPs detected only in a single individual or deviated from Hardy–Weinberg equilibrium (Hardy–Weinberg test, *P *<* *0.05) were excluded from the subsequent analysis (resulting in the exclusion of 23 SNPs and 13 SNPs, respectively). We calculated a normalized coefficient of LD, *D*′ (Falconer 1989), for all pairs of 133 SNPs in a long supercontig (936,214–6,448,156 bp) including the introgression sites on LG17 using Haploview. The reference genome in ENSEMBL sometimes contains misaligned supercontigs (Roesti et al. 2013). Although Roesti et al. (2013) corrected misalignment of some supercontigs, correction is still difficult for small supercontigs. Furthermore, we do not know the actual physical length of gaps between supercontigs. Therefore, we did not use the region from 1 to 936,213 bp, which is composed of four small supercontigs.

SNP pairs were categorized into three types: pairs within the downstream region outside IS1, within IS1 and between IS1 and the downstream region. In each category of the pairs, *D*′ was plotted against the genetic distance (cM) and fitted to the simple model for decay of LD (Falconer 1989): *D*′ = (1 − *θ*)^*t*^, where *θ* is the recombination fraction of two markers, and *t* is the number of generations since *D*′ = 1. For this analysis, genetic distances between the marker pairs were estimated using definite integrals of a Loess curve fitted to the previously reported stickleback recombination rates (cM/Mb) (Roesti et al. 2013). Marker pairs with a distance of 5 cM or less were used. For fitting of LD decay curve, the nls.lm function of the minpack.lm package in R was used (https://cran.r-project.org/web/packages/minpack.lm/index.html).

### Gene sequence analysis

To investigate whether there are any genes with functionally diverged amino acids, we calculated the interallelic *K*_a_/*K*_s_ test of the genes located within the *G. nipponicus* introgression site (1.169–1.622 Mb on LG17) in an heterozygous individual as described previously (Yoshida et al. 2014). Two virtual haploid protein‐coding sequences were reconstructed from the SNP data of one Lake Towada individual with the *G. nipponicus* introgression at IS1 on LG17 (Towada 1). Any indels were masked for the analysis. When more than two heterozygous SNPs exist in a single codon, this method can lead to the reconstruction of the wrong amino acid. In such a case, therefore, we phased heterozygous SNPs manually by checking the short read sequences. The software codeml in PAML 4 (Yang 2007) was used for *K*_a_/*K*_s_ calculation. *K*_a_/*K*_s_ values were calculated using the Nei–Gojobori method (Nei and Gojobori 1986). For genes with *K*_a_/*K*_s_ > 1, rejection of *K*_a_/*K*_s_ = 1 was tested with MEGA6 (Tamura et al. 2013).

To test whether nonsynonymous mutations within IS1 are functionally important, we used Protein Variation Effect Analyzer (PROVEAN) (Choi et al. 2012). Under the assumption that evolutionarily conserved amino acid positions across multiple species are under purifying selection, amino acid substitutions that occurred at highly conserved positions are likely to change protein functions. PROVEAN analysis consists of two steps: collection of orthologs of other species from the NCBI NR protein database, followed by calculation of unbiased averaged delta scores (PROVEAN scores). Delta scores indicate the differences in the alignment scores between a protein query sequence and its variant, therefore reflecting the impacts of the mutation on protein alignment. We calculated the PROVEAN scores of all SNPs that are divergent between species, but are fixed within species in IS1. Significance thresholds for the absolute values of the PROVEAN scores were set at three different values: strict threshold = 4.1 (specificity = 90.3%, sensitivity = 57.6%), middle threshold = 2.5 (specificity = 78.6%, sensitivity = 80.4%), and sensitive threshold = 1.3 (specificity = 61.6%, sensitivity = 90.6%).

## Results

### Genomic regions with high average heterozygosity in the Lake Towada population

Genomewide average heterozygosity (*h*) in the Lake Towada population was 1.633 × 10^−3^, which was close to the value of the Aisaka Pond (1.785 × 10^−3^) but much smaller than that of the anadromous form of *G. aculeatus* (3.097 × 10^−3^). The proportions of heterozygous sites showed the same pattern: values were similar between the Towada (Fig. S2, 1.549 ± 0.150 × 10^−3^) and Aisaka populations (Fig. S2, 1.614 ± 0.065 × 10^−3^), but both were much lower than those of the anadromous population (Fig. S2, 3.120 ± 0.064 × 10^−3^; Mann–Whitney *U*‐test: Towada vs. anadromous, *P* = 0.02857; Aisaka vs. anadromous, *P* = 0.02857). These data suggest that the Lake Towada population did not experience a severe reduction in genetic diversity when it was introduced to Lake Towada but rather that genetic diversity was low in the Aisaka Pond source population.

**Figure 2:**
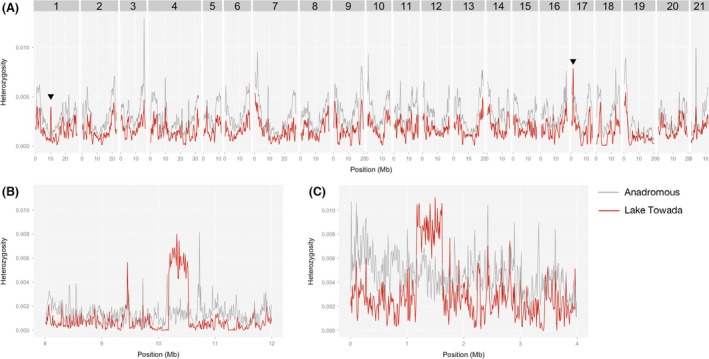
Sliding window analysis of average heterozygosity (*h*) in the genome of Lake Towada population (red line). The average heterozygosity (*h*) of anadromous population is also shown as gray line for comparison. (A) 500‐kb sliding window analysis with 100‐kb step. (B, C) 10‐kb sliding window with 10‐kb shift around the regions with high heterozygosity on LG1 (B) and LG17 (C) of Lake Towada stickleback, which are indicated by triangles in figure A.

To further investigate the genomewide patterns of genetic diversity, we conducted a sliding window analysis of average heterozygosity (*h*) across the genome. Overall patterns of average heterozygosity were similar between the Lake Towada and anadromous populations (Fig. 2A). As expected from the genomewide average heterozygosity, the average heterozygosity of the Lake Towada population was generally lower than that of the anadromous form for most of the genome (Fig. 2A). However, we found two sharp peaks of average heterozygosity on LG1 and LG17 in the Lake Towada population (Fig. 2A), and these peaks were even higher than those in the anadromous population (Fig. 2B and C). The peak on LG17 was the highest across the whole genome of the Lake Towada population (Fig. 2A). Consistent with these data, we found an increase in the proportion of heterozygous sites at this LG17 locus in three of four individuals from the Lake Towada population (Fig. S3).

**Figure 3:**
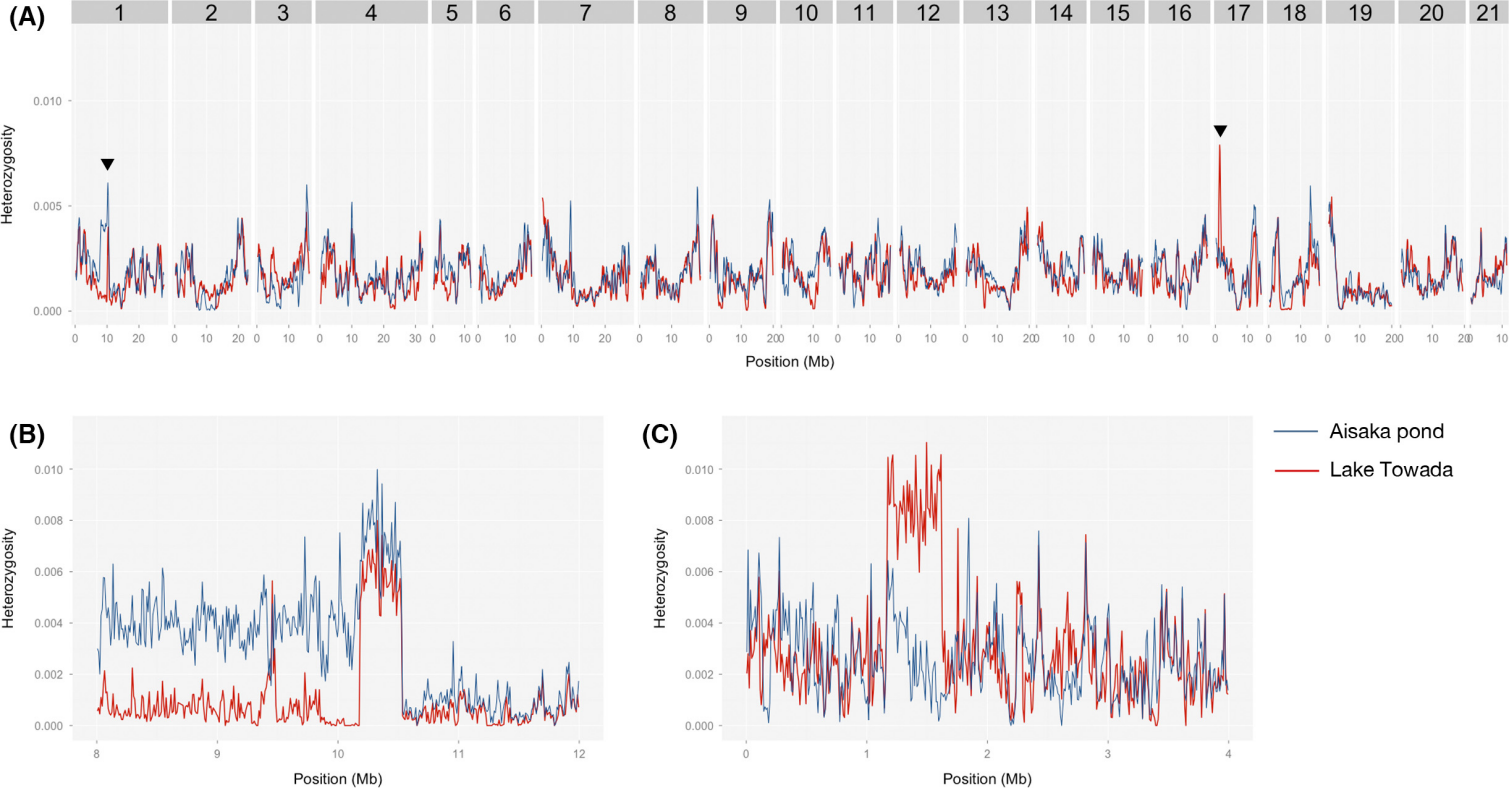
Sliding window analysis of average heterozygosity (*h*) in the genome of Aisaka Pond population (blue line). The average heterozygosity (*h*) of the Lake Towada population is also shown as red line for comparison. (A) 500‐kb sliding window with 100‐kb step. (B, C) 10‐kb sliding window with 10‐kb step around the regions with high heterozygosity on LG1 (B) and LG17 (C) of Lake Towada stickleback, which are indicated by triangles in figure A.

In the Aisaka Pond population, we found a pattern of average heterozygosity similar to that of the Lake Towada population across the genome (Fig. 3A). On LG1, the flanking regions of the peak had rather higher average heterozygosity in the Aisaka Pond stickleback than in the Lake Towada population (Fig. 3B). As for the LG17 peak, the Aisaka Pond population showed no clear peak in average heterozygosity (Fig. 3C). One individual from the Aisaka Pond had an increased proportion of heterozygous sites on LG17, although the size of the region containing the higher proportion of heterozygous sites was smaller than that observed in Lake Towada (see Aisaka3 in Fig. S4). In summary, these data suggest that the Lake Towada population possesses several genomic regions with high average heterozygosity and the ancestral Aisaka population also has a similar pattern of average heterozygosity.

### Introgression of the *G. nipponicus* genome into the Lake Towada population

**Figure 4:**
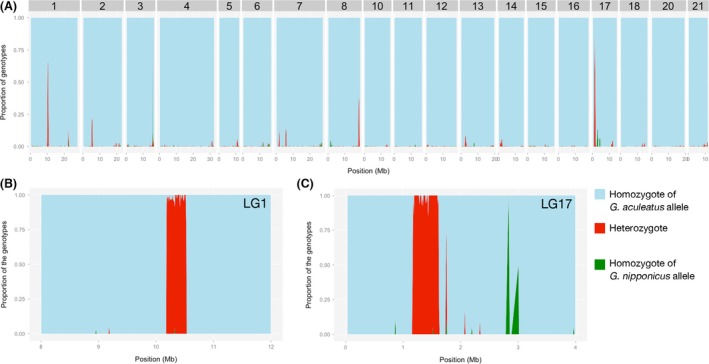
Sliding window analysis of proportions of different genotypes; homozygotes of *G. aculeatus* alleles (light blue), homozygotes of *G. nipponicus* alleles (green), and heterozygotes (red) in one individual from Lake Towada population (Towada 3). (A) 500‐kb sliding window with 100‐kb step. All chromosomes except for sex chromosomes (LG9 and LG19) are shown. (B, C) 10‐kb sliding window with 10‐kb step around the introgression sites on LG1 (B) and LG17 (C).

The proportions of heterozygous sites (i.e., nucleotide differences between two alleles within a single individual) at the regions with high heterozygosity on LG17 in the Lake Towada stickleback (see Towada 1–3 in Fig. S3) were similar to the average substitution rates between *G. aculeatus* and *G. nipponicus* (i.e., nucleotide differences between the two species) (0.0144; Yoshida et al. 2014). Therefore, we hypothesized that regions with high heterozygosity may result from hybridization between the two species. To test this hypothesis, we calculated the proportions of sequences derived from *G. nipponicus* for each individual from Lake Towada. To perform this analysis, we calculated the proportion of *G. nipponicus*‐derived sequences in the Lake Towada population (see “Materials and Methods”). A genomewide sliding window analysis of the proportion of *G. nipponicus*‐derived sequences revealed 19 regions longer than 10 kb with genotypes with *G. nipponicus*‐specific alleles within the genome of the Lake Towada stickleback (Figs. 4A, S5). A 10‐kb sliding window analysis on LG1 and LG17 showed that regions with high average heterozygosity (see the above section) correspond to the regions where *G. nipponicus* alleles were found (Figs. 4B, C, S6). One potential introgression site on LG17 had a sharp boundary at 1,169,000 and 1,622,000 bp. These data suggest that high average heterozygosity in this region resulted from introgression of *G. nipponicus* alleles.

To examine whether introgression sites of this size would be detectable by two recently developed and commonly used reduced‐representation methods, RAD and ddRAD sequencing technologies, we conducted in silico RAD and ddRAD sequencing (Fig. S1). Under the assumption that all restriction sites can be perfectly sequenced at high coverage in RAD and ddRAD sequencing, we counted the number of detectable *G. nipponicus*‐specific SNPs (Table S2). The number of informative SNPs decreased in both RAD and ddRAD sequencing compared to whole‐genome sequencing. More than half of the introgression sites lack any informative SNPs, suggesting that these introgression sites would be overlooked even if RAD and ddRAD sequencing worked perfectly. In the case of ddRAD sequencing with SphI‐EcoRI, only two SNPs would be captured.

**Figure 5:**
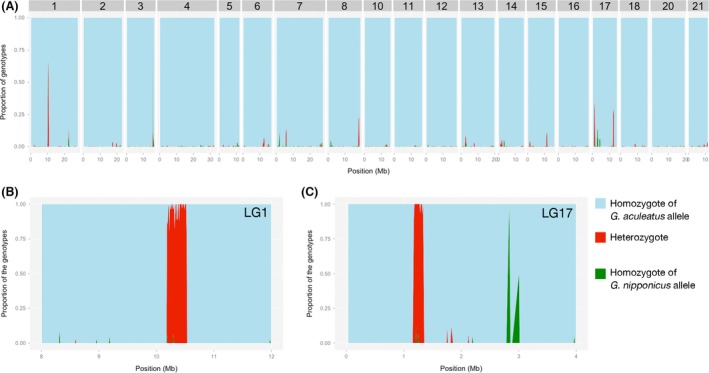
Sliding window analysis of proportions of different genotypes; homozygotes of *G. aculeatus* alleles (light blue), homozygotes of *G. nipponicus* alleles (green), and heterozygotes (red) in one individual from Aisaka Pond (Aisaka3). (A) 500‐kb sliding window with 100‐kb step. All chromosomes except for sex chromosomes (LG9 and LG19) are shown. (B, C) 10‐kb sliding window with 10‐kb step around the introgression site on LG1 (B) and LG17 (C).

The genomewide sliding window analysis of the Aisaka Pond stickleback genome sequences indicated that the Aisaka Pond stickleback had 23 regions longer than 10 kb with genotypes with *G. nipponicus* alleles (Figs. 5A, S7). Sixteen introgression sites found in the genomes of the Lake Towada population (84%) overlapped with the introgression sites identified in the Aisaka Pond population. A high proportion of genotypes with *G. nipponicus* alleles was also observed on LG1 (Figs. 5B, S8A). On LG17, one individual that exhibited a high proportion of heterozygous sites on LG17 was a heterozygote of *G. aculeatus* and *G. nipponicus* alleles at this locus, although the region with the high proportion of heterozygous sites and introgression was narrower (1.169–1.346 Mb) than the region observed in the Lake Towada population (Figs. 5C, S8B).

### LD around the introgression site

To further investigate the introgression site IS1 (1.169–1.622 Mb) on LG17 in the Lake Towada population, we conducted amplicon sequencing of 48 genomic regions within and around the introgression site for 48 individuals.

First, the *G. nipponicus* allele frequencies among the 48 individuals were calculated around the introgression site (Fig. S9). As expected, the *G. nipponicus* allele frequency was high within IS1: five amplicons with a high frequency of *G. nipponicus*‐derived alleles are found within IS1 (1.169–1.622 Mb; Fig. S9). There are some variations in the *G. nipponicus* allele frequency among five amplicons, which may result from recombination within IS1. There was another locus with a high frequency of *G. nipponicus*‐derived alleles (termed IS2; Fig. S9), which is located next to IS1 and corresponds to a short introgression site (1.749–1.759 Mb; Fig. 4C). Other flanking regions had no *G. nipponicus* alleles. Hardy–Weinberg equilibrium tests indicated that no SNPs at these *G. nipponicus* diagnostic loci deviated from Hardy–Weinberg equilibrium (exact test, *P *>* *0.05).

**Figure 6:**
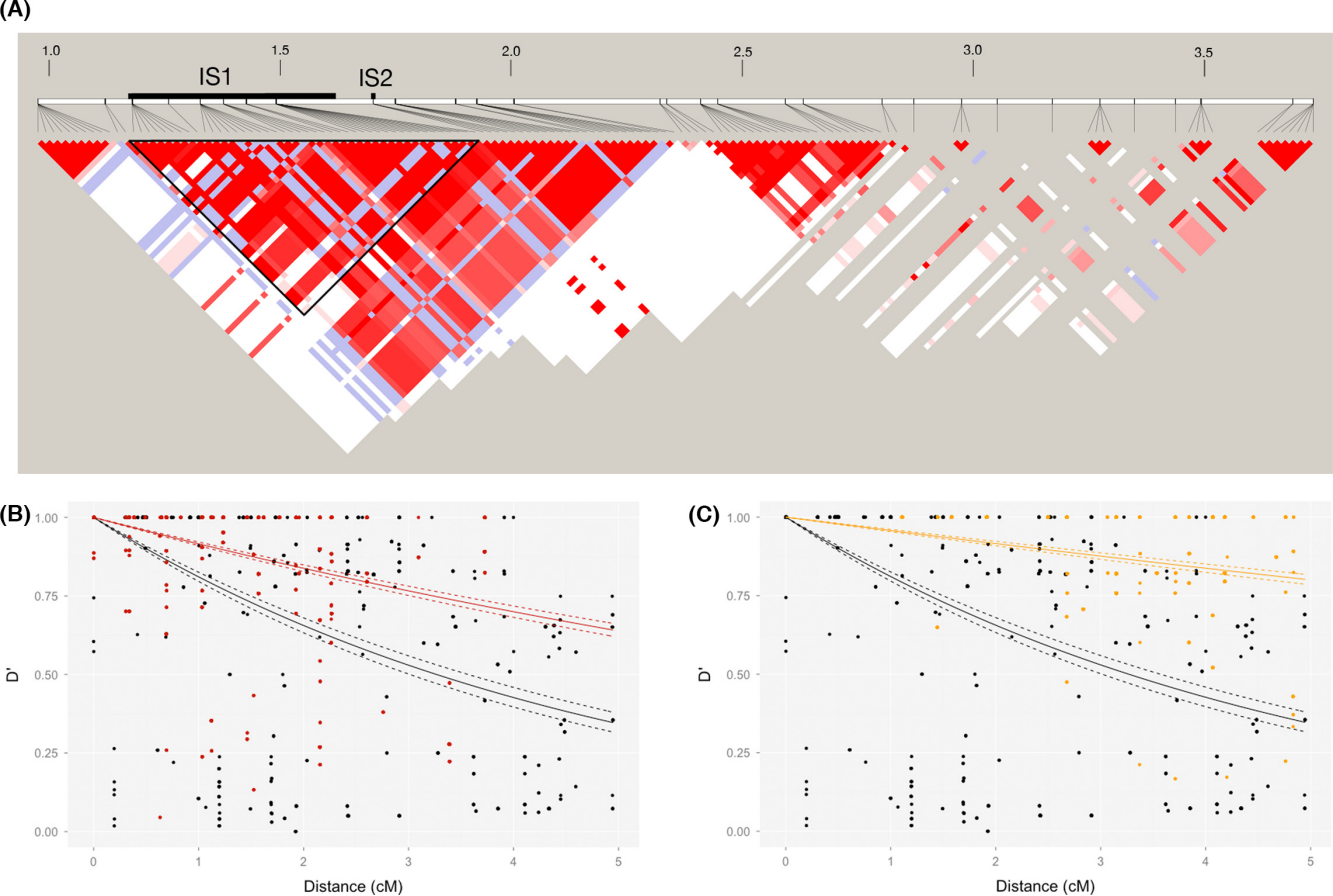
(A) Linkage disequilibrium (*D*′) around the introgression site on LG17. Color intensity indicates the value of *D*′. Red colors indicate LOD ≥2, whereas blue colors indicate LOD < 2. Bold black lines above the LD data indicate two introgression sites, IS1 and IS2. The triangle surrounded by a bold black line indicates LD within IS1. The numbers on the panel indicate the position on LG17 in Mb. Only LD between loci with the distance of <1 Mb are shown here. (B) LD decays more slowly within the introgression site IS1 (red) than outside IS1 (black). (C) Decay of LD between a marker within IS1 and a marker outside IS1 (orange) was also slower than the LD decay between markers outside IS1 (black). Dotted lines indicate 95% confidence intervals.

Next, we conducted LD analysis (Fig. 6). If the introgression occurred and spread recently, there would be little time for recombination to break down LD, so high LD around the introgression site should be observed. For LD analysis, we used 133 nucleotide loci on a large supercontig (from 936,214 to 6,448,157 bp) including both IS1 and IS2. We did not use the genomic region from 1 to 936,213 bp on LG17 because the public genome sequence of this region is composed of four supercontigs in the ENSEMBL database, so we cannot exclude the possibility that these supercontigs were misaligned in the database (see the “Materials and Methods”).

We found a high LD not only between markers within the introgression site IS1 but also between a marker within IS1 and a maker in the nearby flanking regions (Fig. 6A). Then, we calculated the decay of LD for marker pairs inside IS1, marker pairs outside IS1, and pairs between one marker inside and another outside IS1. LD decays more slowly within the introgression site (red in Fig. 6B) than outside the introgression site (black in Fig. 6B). More importantly, LD also decays slowly between a marker inside IS1 and a marker outside IS1 (orange in Fig. 6C).

### Potential functional differences between *G. aculeatus* and *G. nipponicus* alleles at the introgression site

The introgression site IS1 contained 38 protein‐coding genes. To investigate whether these genes are functionally different between the *G. nipponicus* and *G. aculeatus* alleles, we first conducted interallelic *K*_a_/*K*_s_ analysis. Although there are few genes with *K*_a_/*K*_s_ > 1 between *G. nipponicus* and *G. aculeatus* alleles (possibly due to the recent divergence of the two species), two genes with *K*_a_/*K*_s_ > 1 were found: a pentraxin‐like novel gene (ENSGACG00000003825; *K*_a_/*K*_s_ = ∞, *K*_a_ = 0.0143, *K*_s_ = 0) and a coiled‐coil domain‐containing protein 66‐like novel gene (ENSGACG00000003880; *K*_a_/*K*_s_ = ∞, *K*_a_ = 0.0049, *K*_s_ = 0). The pentraxin‐like gene had a significantly higher *K*_a_/*K*_s_ than 1 (*P* = 0.002), although *K*_a_/*K*_s_ > 1 was not significant for the latter gene (*P *>* *0.05).

Next, we conducted PROVEAN analysis, which screens for functionally important mutations by searching for nucleotide changes that occurred at phylogenetically conserved nucleotides. We found several genes with high PROVEAN scores (Table 2): 13 genes with a sensitive threshold, five genes with a middle‐level threshold, and three genes with a strict threshold (see the “Materials and Methods”). One gene, filamin B‐like gene, had three amino acid substitutions with high PROVEAN scores (−2.963 for Asp to Glu substitution at the amino acid position 148; 6.973 for Ala to Pro substitution at the position 938; 3.165 for Gly to Ala at the position 2464).

**Table 2:** Possible functional substitutions between *G. nipponicus* and *G. aculeatus* alleles within the introgression site IS1

Gene postion^1^	Gene name	Transcript ID	Amino acid change [PROVEAN Score^2^]
1,175,845	Transglutaminase 8	ENSGACT00000004923	V526A [1.373]
1,200,852	Filamin B, like	ENSGACT00000004976	**D148E** [−**2.963** *], **A938P** [**6.973** **], N1851S [1.58]
		ENSGACT00000004983	K1879E [−2.084], **G2464A** [**3.165** *]
1,235,360	Sarcolemma‐associated protein a	ENSGACT00000005008	N813S [−1.396]
1,281,299	Kelch‐like family member 10 [2/5]	ENSGACT00000005082	**D178E** [**2.7** *], R415P [−1.733], T505I [−1.647]
		ENSGACT00000005084	K229Q [−1.76], Q479H [−2.241]
1,343,836	Coiled‐coil domain‐containing 66	ENSGACT00000005108	**L189R** [−**4.579** **]
1,355,299	Inositol hexakisphosphate kinase 1	ENSGACT00000005112	P375R [−1.362]
1,439,329	Potassium channel tetramerisation domain‐containing 6a	ENSGACT00000005237	S4C [−1.661]
1,465,445	Stabilin 1 [1/2]	ENSGACT00000005255	Q577K [1.418], K760R [2.164]
1,482,835	Stabilin 1 [2/2]	ENSGACT00000005273	S380L [−2.495], T494P [1.338]
1,499,288	Nischarin	ENSGACT00000005292	**S298G** [−**3.199** *], I569N [−1.335], C1080R [1.605]
1,563,301	Asporin (LRR class 1)	ENSGACT00000005358	P18H [1.458]
1,578,802	Osteoglycin	ENSGACT00000005363	L211M [−1.745]
1,593,763	Nucleolar protein 8	ENSGACT00000005381	**E308G** [−**6.398** **]

Genes with absolute values of PROVEAN scores > 1.3 are listed here. ^1^Median position of genes, ^2^amino acid changes are shown by the amino acid in *G. aculeatus*, amino acid position, and the amino acid in *G. nipponicus*. Positive and negative PROVEAN scores indicate that functional mutation occurred in *G. aculeatus* and *G. nipponicus*, respectively. *PROVEAN scores > 2.5 or < −2.5. **PROVEAN scores > 4.1 or < −4.1. Bold letters indicate PROVEAN scores whose absolute values are larger than 2.5.

## Discussion

### Small genomic regions of introgression detected with whole‐genome sequencing

By conducting whole‐genome sequencing of four individuals of an introduced population with high coverage, we identified many small genomic regions of interspecies introgression (Figs. 4, S5). These introgressions could not be detected using our previous microsatellite analysis (Adachi et al. 2012). Although reduced‐representation sequencing, such as RAD and ddRAD sequencing, is a good alternative to whole‐genome sequencing, even these methods risk overlooking introgression sites: More than half of introgression sites >10 kb would have been overlooked (Table S2). Furthermore, the number of conserved restriction sites decreases with increasing genetic distance between parental species (Gautier et al. 2013). In our in silico analysis, we assumed that all of the restriction sites can be perfectly sequenced, but this is not usually the case, suggesting that more SNPs than expected within the introgression sites would be overlooked in real RAD and ddRAD experiments. Thus, our results exemplify a case that introduced populations can have small regions of introgression that can be detected with whole‐genome sequencing but can be overlooked with reduced‐representation sequencing. Instead, marker‐based analysis and reduced‐representation sequencing are more useful to increase sample size for testing hypotheses formulated from whole‐genome sequence data. For example, we used targeted amplicon sequencing of 48 individuals, a reduced‐representation method, to test a selective sweep of one introgression site (Fig. 6).

### Potential roles of introgression in colonization of novel environments

Hybridization increases genetic variation and helps invasive species adapt to novel environments (Lee 2002; Dlugosch and Parker 2008; Prentis et al. 2008). The Lake Towada population may be another such example. Several peaks of high heterozygosity in the Lake Towada population resulted from interspecies introgressions. If these regions resulted from shared polymorphism between species, we would see a similar increase in average heterozygosity in the anadromous population, which we did not observe. LD analysis also supports the idea that these regions are derived from introgression. Our data also indicate that the ancestral Aisaka Pond population already possessed the introgression site. This data indicates the possibility that the two species were introduced together to the pond on the trout farm, hybridized there, and were introduced to Lake Towada, although we cannot exclude the alternative possibility that progeny of natural hybrids formed in a sympatric habitat were introduced into the trout farm. Environmental changes and artificial environments are thought to reduce the ecological disadvantage of hybrids and promote hybridization between species (Seehausen et al. 2008), and the trout farm may be one of such artificial environments.

Several data are consistent with the hypothesis that the introgression was adaptive. First, LD analysis showed high LD between the introgression site and the flanking region on LG17 (Fig. 6), and one possible explanation for high LD is the genetic hitchhiking effect of directional or balancing selection on the introgression site. Although another possible explanation for high LD is recombination suppression, this is unlikely. While the IS1 on LG17 was 450 kb long in three Lake Towada individuals (Figs. 4B, S6), the introgression site was shorter in one individual from the Aisaka Pond population (Figs. 5B, S8). In addition, variations in the *G. nipponicus* allele frequencies were found among amplicons within IS1 (Fig. S9). These data indicate that recombination can occur even within IS1. Second, our gene sequence analysis showed that the introgression site may have genes with functional changes between *G. aculeatus* and *G. nipponicus* alleles. For example, the Filamin B‐like gene contained multiple amino acid substitutions that are potentially functional. Filamin B is a cytoplasmic protein involved in skeletal development (Lu et al. 2007). Another interesting gene within the introgression site was a Pentraxin‐like gene with *K*_a_/*K*_s_ > 1. This gene may be under divergent selection between two species. Although we do not know the function of this gene, pentraxin domain‐containing genes are generally activators of the innate immune system and bound to pathogens and damaged cells (Bottazzi et al. 2006).

In conclusion, whole‐genome sequencing of even a small number of individuals can provide information about the history of past hybridization and candidate genes important for adaptation, which may be overlooked using traditional marker‐based analysis. Therefore, the use of a combination of whole‐genome sequencing technologies and marker‐based analyses or reduced‐representation sequencing would be a powerful way to understand the demographic history and candidate genes of introduced populations.

## Conflict of Interest

None declared.

## Data Archiving Statement

All sequence data will be available from DDBJ after acceptance.

## Supporting information


**Figure S1.** Schematic diagram of in silico RAD with SbfI (A) and ddRAD sequencing with EcoRI‐MspI (B) are shown.

**Figure S2**. The average proportion of heterozygous sites across the whole genome.

**Figure S3**. Sliding window analysis (10‐kb sliding window with 10‐kb step) of the proportion of heterozygous sites on LG17 in four individuals of Lake Towada stickleback (Towada1–Towada4).

**Figure S4**. Sliding window analysis (10‐kb sliding window with 10‐kb step) of the proportion of heterozygous sites on LG17 in multiple individuals of Aisaka pond stickleback (Aisaka1–Aisaka4).

**Figure S5.** Sliding window analysis (500‐kb sliding window with 100‐kb step) of proportion of different genotypes; homozygotes of *G. aculeatus* alleles (light blue), homozygotes of *G. nipponicus* alleles (green), and heterozygotes (red) in four individuals from Lake Towada (Towada1–Towada4).

**Figure S6**. Sliding window analysis (10‐kb sliding window with 10‐kb step) of proportion of different genotypes around the regions with high heterozygosity on LG1 (A) and LG17 (B); homozygotes of *G. aculeatus* alleles (light blue), homozygotes of *G. nipponicus* alleles (green), and heterozygotes (red) in four individuals from Lake Towada (Towada1–Towada4).

**Figure S7.** Sliding window analysis (500‐kb sliding window with 100‐kb step) of proportion of different genotypes; homozygotes of *G. aculeatus* alleles (light blue), homozygotes of *G. nipponicus* alleles (green), and heterozygotes (red) in four individuals from Aisaka Pond (Aisaka1–Aisaka4).

**Figure S8.** Sliding window analysis (10‐kb sliding window with 10‐kb step) of proportion of different genotypes around the regions with high heterozygosity on LG1 (A) and LG17 (B); homozygotes of *G. aculeatus* alleles (light blue), homozygotes of *G. nipponicus* alleles (green), and heterozygotes (red) in four individuals from Aisaka Pond (Aisaka1–Aisaka4).

**Figure S9.** Frequency of *G. nipponicus*‐specific alleles within and around the introgression sites on LG17.

**Table S1.** The amplified region and primers used in the amplicon sequencing of Lake Towada population.

**Table S2.** Number of *G. nipponicus*‐specific SNPs detectable in the introgression sites in the 19 introgression sites by whole genome sequencing (WGS), simulated RAD sequencing and simulated ddRAD sequencing.
