# Supplementary figures and images for: Whole‐genome sequencing reveals small genomic regions of introgression in an introduced crater lake population of threespine stickleback
Source: Ecol Evol. 2016 Mar 2;6(7):2190–204. doi: 10.1002/ece3.2047 (PMC4782248; doi:10.1002/ece3.2047)

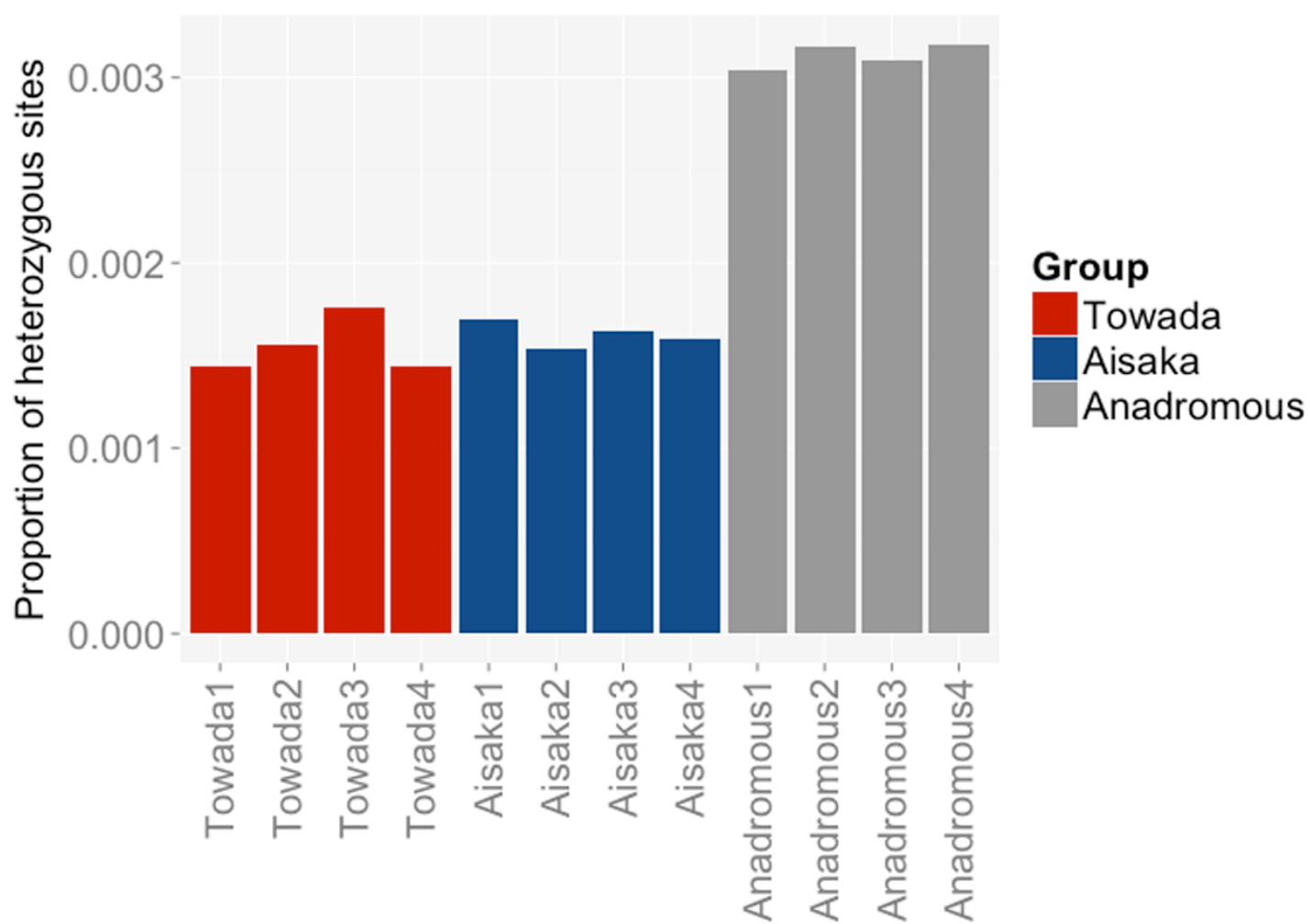

Supplement: Supplementary file 2 — Figure S2. The average proportion of heterozygous sites across the whole genome. [file ECE3-6-2190-s002.pdf]

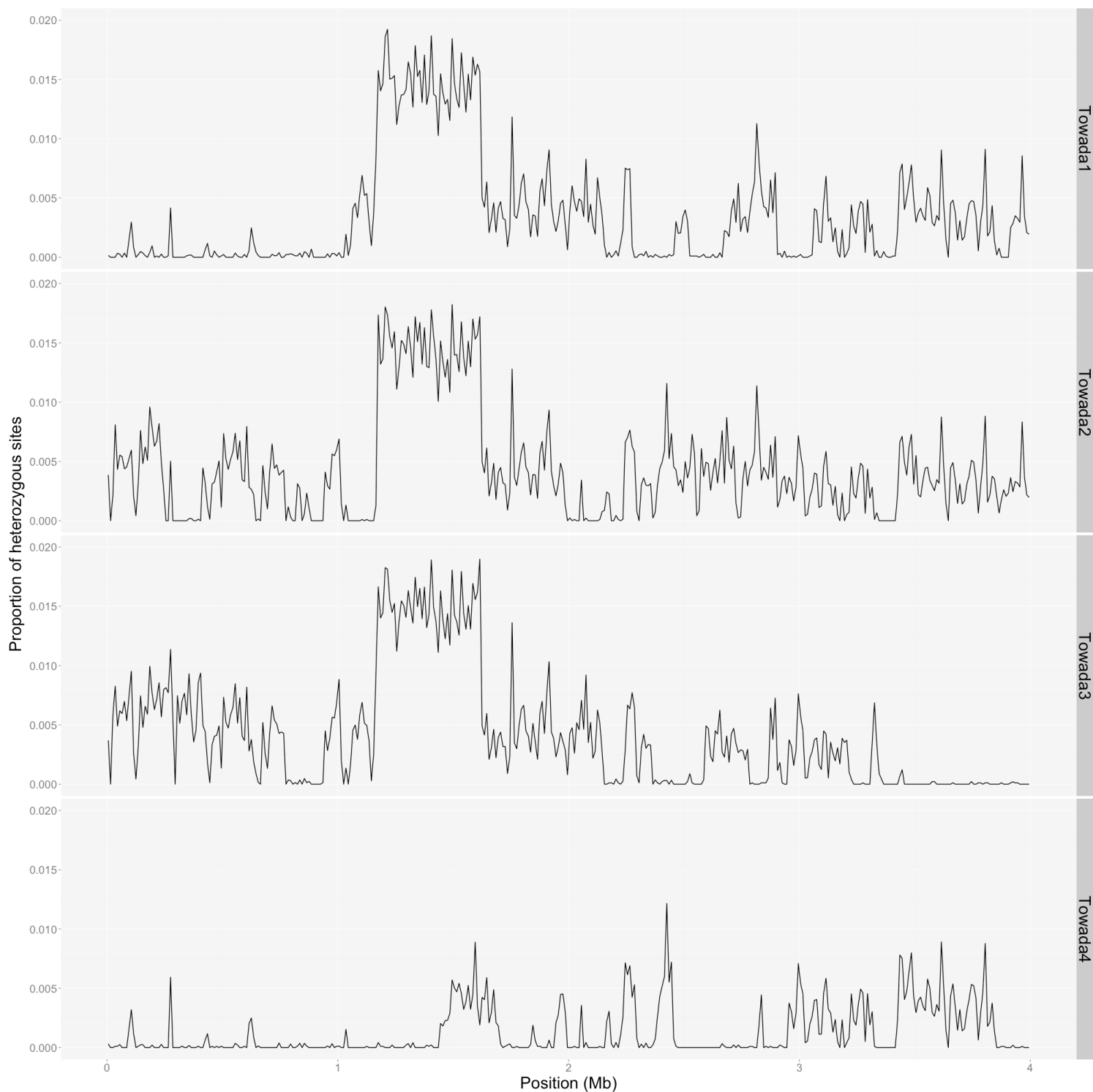

Supplement: Supplementary file 3 — Figure S3. Sliding window analysis (10‐kb sliding window with 10‐kb step) of the proportion of heterozygous sites on LG17 in four individuals of Lake Towada stickleback (Towada1–Towada4). [file ECE3-6-2190-s003.pdf]

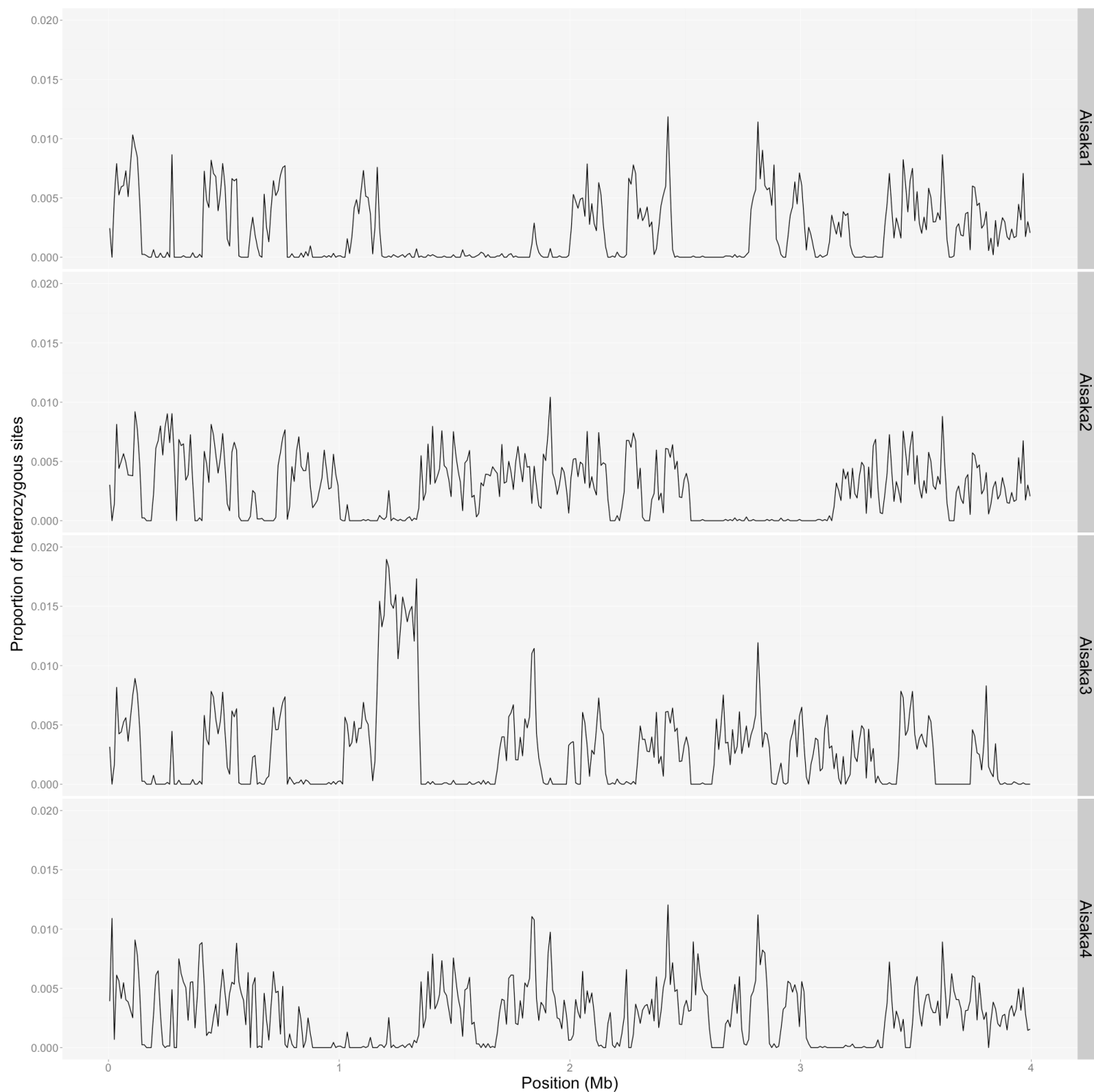

Supplement: Supplementary file 4 — Figure S4. Sliding window analysis (10‐kb sliding window with 10‐kb step) of the proportion of heterozygous sites on LG17 in multiple individuals of Aisaka pond stickleback (Aisaka1–Aisaka4). [file ECE3-6-2190-s004.pdf]

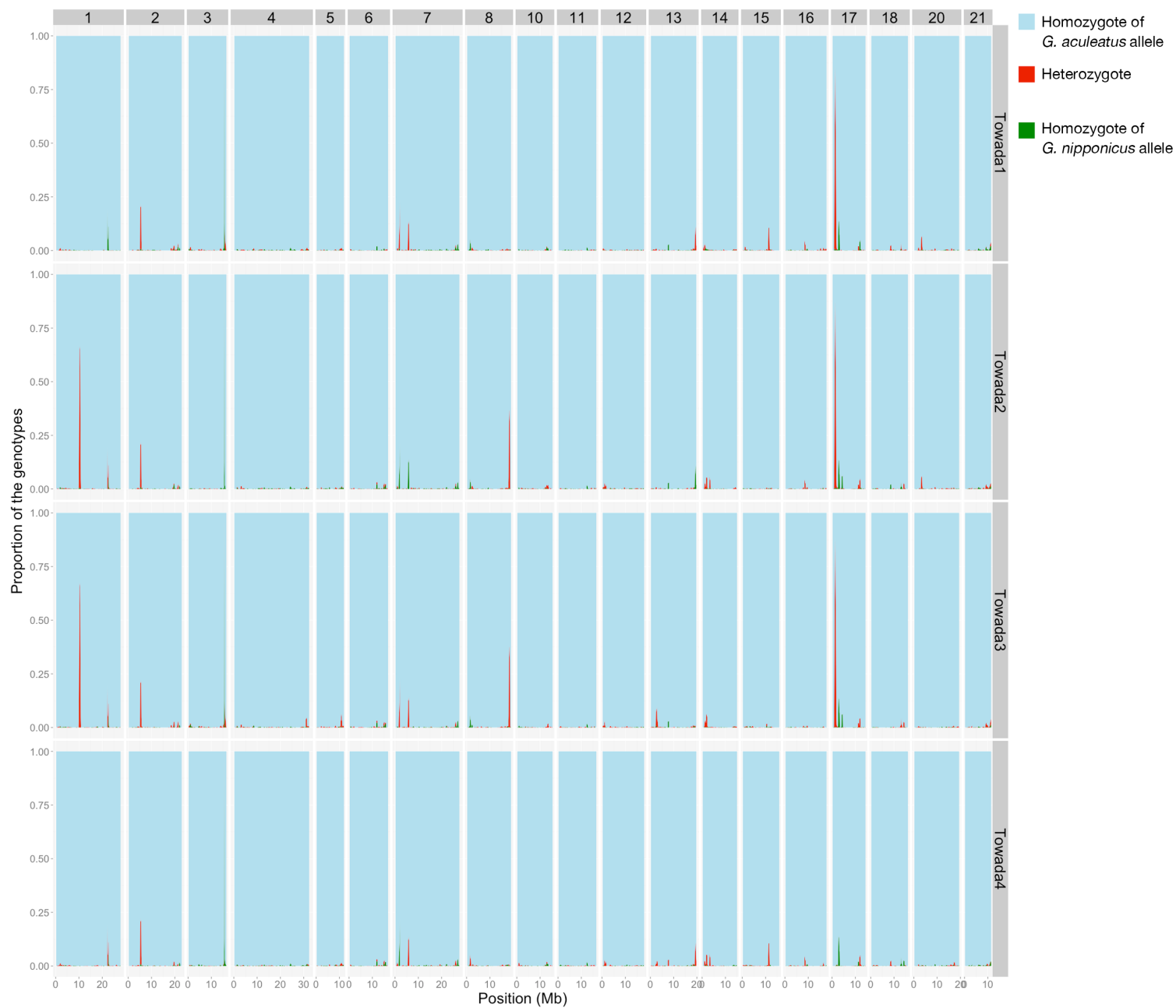

Supplement: Supplementary file 5 — Figure S5. Sliding window analysis (500‐kb sliding window with 100‐kb step) of proportion of different genotypes; homozygotes of G. aculeatus alleles (light blue), homozygotes of G. nipponicus alleles (green), and heterozygotes (red) in four individuals from Lake Towada (Towada1–Towada4). [file ECE3-6-2190-s005.pdf]

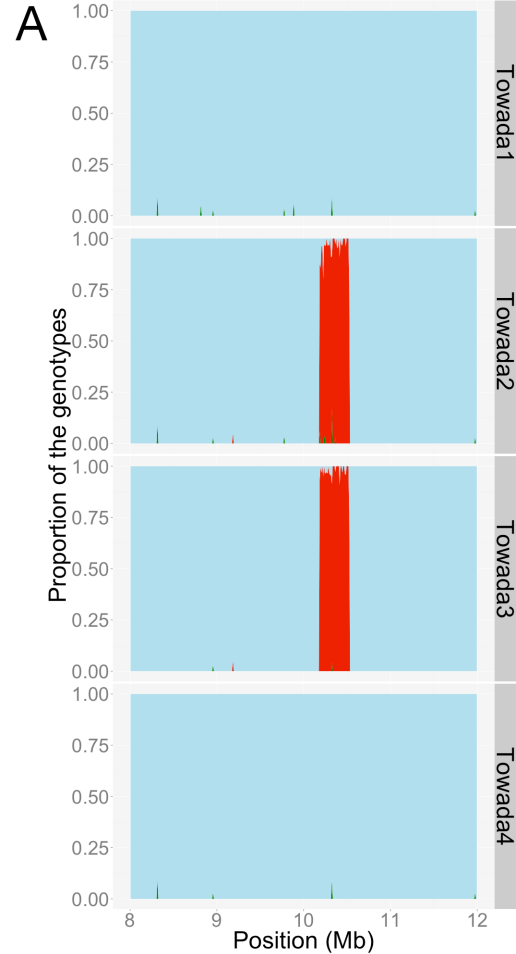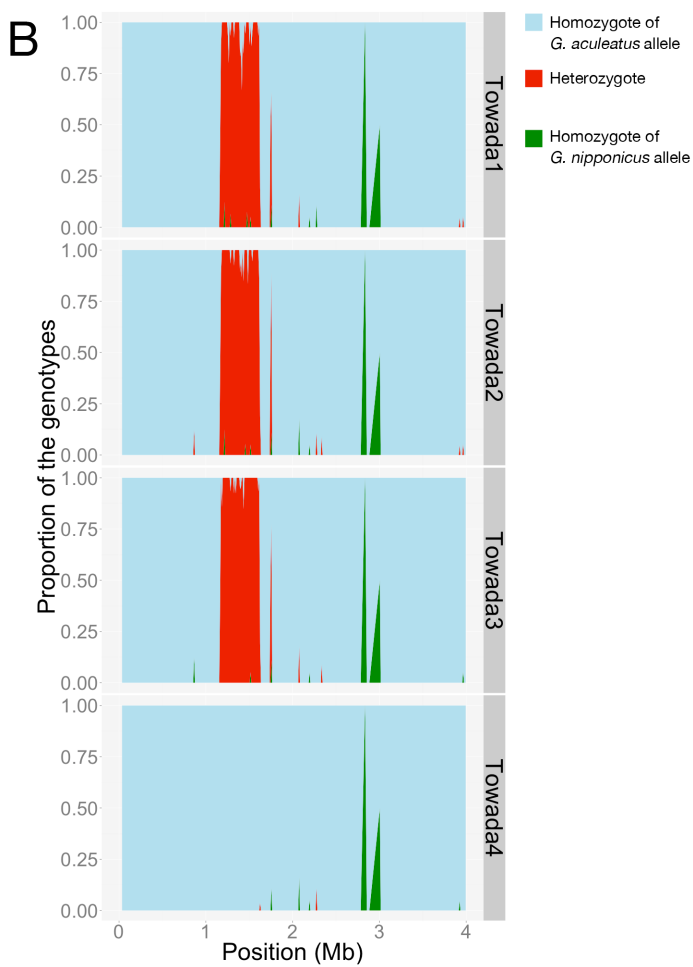

Supplement: Supplementary file 6 — Figure S6. Sliding window analysis (10‐kb sliding window with 10‐kb step) of proportion of different genotypes around the regions with high heterozygosity on LG1 (A) and LG17 (B); homozygotes of G. aculeatus alleles (light blue), homozygotes of G. nipponicus alleles (green), and heterozygotes (red) in four individuals from Lake Towada (Towada1–Towada4). [file ECE3-6-2190-s006.pdf]

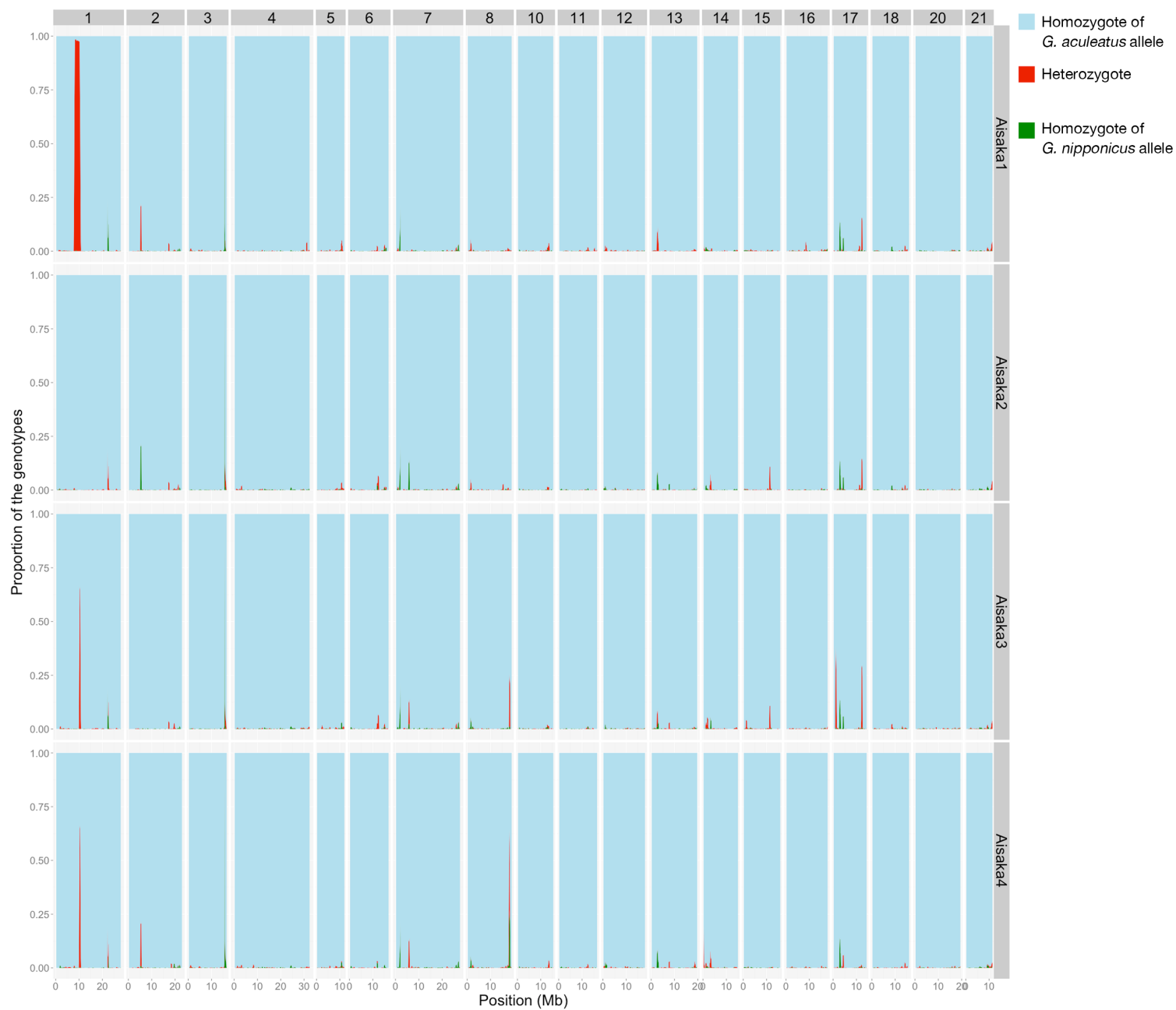

Supplement: Supplementary file 7 — Figure S7. Sliding window analysis (500‐kb sliding window with 100‐kb step) of proportion of different genotypes; homozygotes of G. aculeatus alleles (light blue), homozygotes of G. nipponicus alleles (green), and heterozygotes (red) in four individuals from Aisaka Pond (Aisaka1–Aisaka4). [file ECE3-6-2190-s007.pdf]

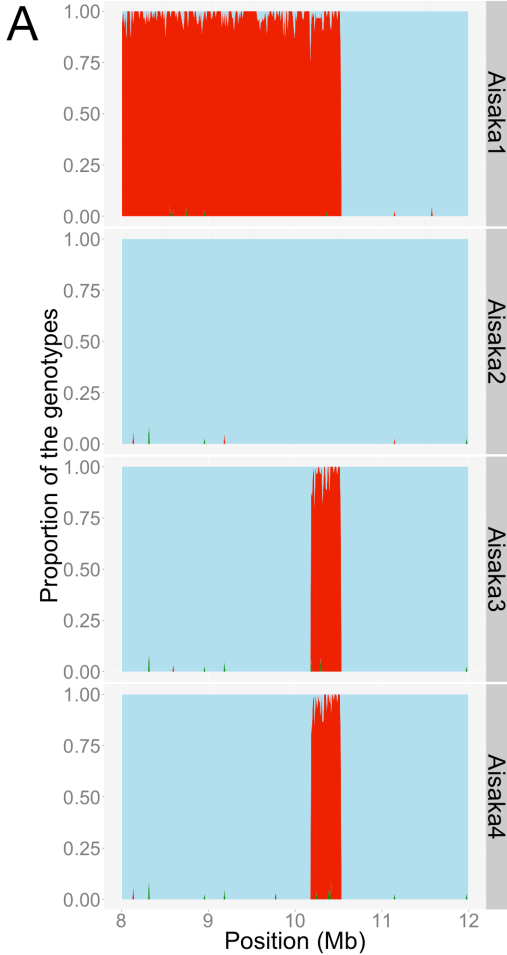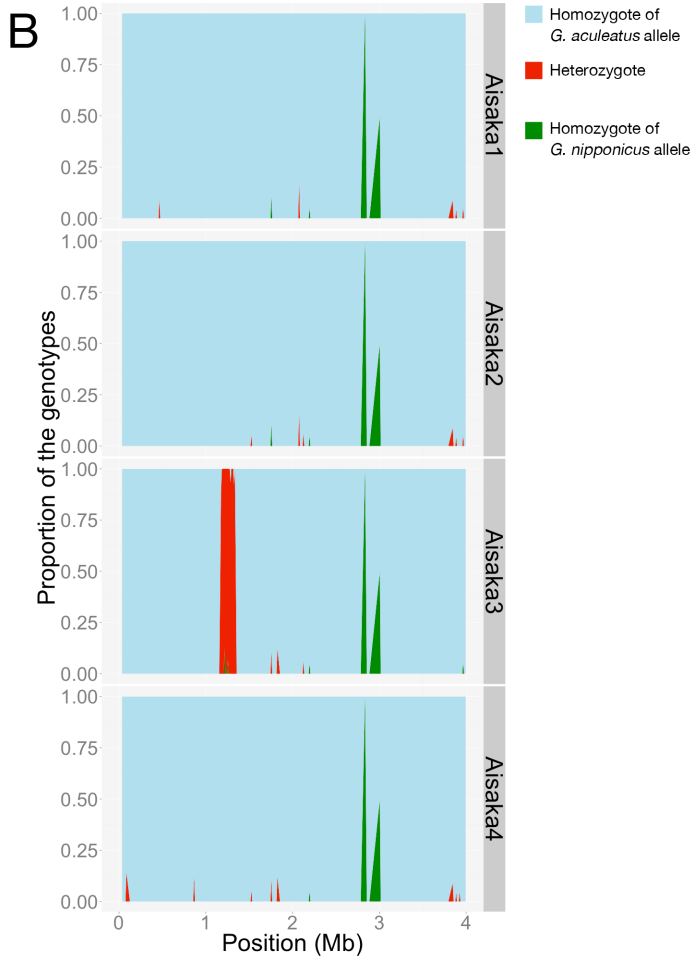

Supplement: Supplementary file 8 — Figure S8. Sliding window analysis (10‐kb sliding window with 10‐kb step) of proportion of different genotypes around the regions with high heterozygosity on LG1 (A) and LG17 (B); homozygotes of G. aculeatus alleles (light blue), homozygotes of G. nipponicus alleles (green), and heterozygotes (red) in four individuals from Aisaka Pond (Aisaka1–Aisaka4). [file ECE3-6-2190-s008.pdf]

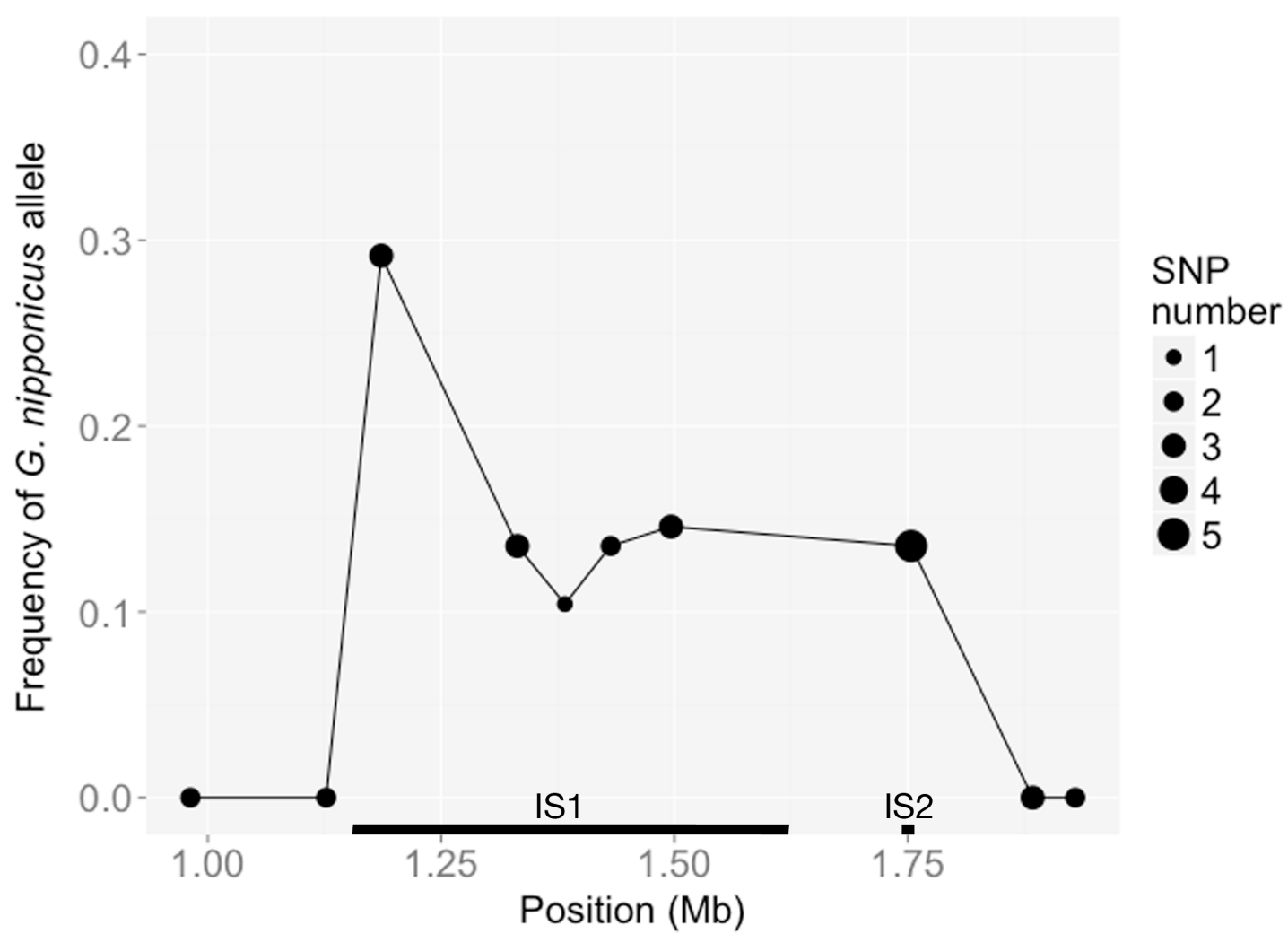

Supplement: Supplementary file 9 — Figure S9. Frequency of G. nipponicus‐specific alleles within and around the introgression sites on LG17. [file ECE3-6-2190-s009.pdf]
