# Supplementary material for: Whole‐genome sequencing reveals small genomic regions of introgression in an introduced crater lake population of threespine stickleback
Source: Ecol Evol. 2016 Mar 2;6(7):2190–204. doi: 10.1002/ece3.2047 (PMC4782248; doi:10.1002/ece3.2047)
Supplement: Supplementary file 10 — Table S1. The amplified region and primers used in the amplicon sequencing of Lake Towada population. [file ECE3-6-2190-s010.docx]

Supplemental table 1. The amplified region and primers used in the amplicon sequencing of Lake Towada population

| Start^*^ | End^*^ | Name | Sequence |
| --- | --- | --- | --- |
| 49183 | 49918 | GACG3035F | GCTGCTCTTTGATTGGTTCC |
|  |  | GACG3035R | TCCATGTACGGACTGTTCCA |
| 139508 | 140171 | GACG3043F | CCACAGGAAGTGACCGATCT |
|  |  | GACG3043R | GGCTCCAACAGGAGAACTTG |
| 318578 | 319247 | GACG3059F | TTCCTGACCGGAGTTCAGAG |
|  |  | GACG3059R | TTGGAGGTTACACCTTGCATC |
| 380136 | 380720 | GACG3062F | ACCGTCCCAAATATCAGCAG |
|  |  | GACG3062R | GACAGGCTTCTCGTTGGAAG |
| 466173 | 466932 | GACG3203F | CGGAGGAGTAGAAGCCATTG |
|  |  | GACG3203R | AAGCTGGACTTCCTGGTCAA |
| 546854 | 547404 | GACG3387F | TGACCTCCTGTTATCTGACTCCT |
|  |  | GACG3387R | ACATTCAAGGGCTCGACATT |
| 635642 | 636467 | GACG3484F | GGAGACATCGTCAGCTGTTTG |
|  |  | GACG3484R | CTTTCACCAGGTCTGCAGTG |
| 705154 | 705763 | GACG3495F | ATCCACCTGTCGTTTCCAAG |
|  |  | GACG3495R | AGGCGCTCATCATTTCTGAC |
| 835204 | 835775 | GACG3559F | CCGTAGAGGTGGAGCTGAAG |
|  |  | GACG3559R | ATTGCGTCCAGACTCCTGTC |
| 980727 | 981544 | GACG3594F | CACGTCATCACAGGTGTTACAG |
|  |  | GACG3594R | AAAGACCTGAAAGCCGTCAC |
| 1101394 | 1102190 | GACG3641F | GGAGTTGCTCATGACGAAGG |
|  |  | GACG3641R | GCAAGTTCGACAACCTGTACG |
| 1126277 | 1126931 | GACG3697F | ACCCTTTCCTTTCCTTCCAC |
|  |  | GACG3697R | GTTGTAAAGACCCTCCTCAAATAAA |
| 1185324 | 1185924 | GACG3768F | ACACACAGCCTAAGACATTGACA |
|  |  | GACG3768R | AACCTGCAGTACAACGTCAGC |
| 1263638 | 1264298 | GACG3825F | AACGCGTCAGTCGTCTCTTT |
|  |  | GACG3825R | GGTGAGCCTGTGATGGACTT |
| 1331513 | 1332209 | GACG3877F | GATGTTCCCTGGCCACTCTA |
|  |  | GACG3877R | CGAGTCGGAGAACCTCAAAG |
| 1382448 | 1383211 | GACG3914F | GGACTGCGAGTTCATCTGGT |
|  |  | GACG3914R | TCCAGAGCTGGAGTCAGACA |
| 1431211 | 1431816 | GACG3959F | AAGACATCATCGCCTCCATC |
|  |  | GACG3959R | TGCTGCAGCTACAATAAATATTCC |
| 1495687 | 1496512 | GACG4013F | CTGGTTCTCCTCCACCTGAC |
|  |  | GACG4013R | CTGTAACCAGGAGGCGTCAC |
| 1599965 | 1600711 | GACG4094F | GCACGCCTCTCTGTCATTTC |
|  |  | GACG4094R | GCAAACAGCAGGAACTGTCC |
| 1705270 | 1705903 | GACG4147F | CCAGTTGTAACAGCCCAGGT |
|  |  | GACG4147R | GAGCCATGCATCTACGACAA |
| 1753093 | 1753777 | GACG4232F | CTTCTCCATGCGGTTTGAAT |
|  |  | GACG4232R | GAACCTCCTGCAAGAACTCG |
| 1808494 | 1809338 | GACG4291F | CCTGCCAGGTGAGTTCAAGT |
|  |  | GACG4291R | CGATCAGCATGGTGTACAGG |
| 1883475 | 1884039 | GACG4342F | CGAAGCCAAACACCTCAACT |
|  |  | GACG4342R | GAAGGTCGTCTTTGTTTATCGTTC |
| 1929225 | 1929898 | GACG4389F | TTCCTGGAGTTCGAGGGTTA |
|  |  | GACG4389R | AGTGCCGAACTTGAGGATGT |
| 2009228 | 2009850 | GACG4412F | TCACTGTGACCAATGTGAAATACA |
|  |  | GACG4412R | CCCTCTGGGTAGGAGAGCAT |
| 2225862 | 2226439 | GACG4448F | TTCACCCAGGTCCTTTCACT |
|  |  | GACG4448R | GAACTCTTTGGCCAGGTGAC |
| 2339825 | 2340416 | GACG4496F | GATTGTTTGGTGGAGCGTTT |
|  |  | GACG4496R | AAGTTCTCGGTGAGGGACTG |
| 2412420 | 2412998 | GACG4509F | TAAACGACACCGTTCTGTGG |
|  |  | GACG4509R | GTGATGCTGCCAGCTTTCTT |
| 2448997 | 2449625 | GACG4517F | CGTTCCCATATATGATGGAGGT |
|  |  | GACG4517R | CCAGCCTGACTGAGCAATAA |
| 2526560 | 2527170 | GACG4633F | GAGGTGCATGCAAAGAGTCA |
|  |  | GACG4633R | ATTTGCACTCACGAGCCTCT |
| 2595221 | 2595822 | GACG4738F1 | GCGGAGTGTCTGATGAAACC |
|  |  | GACG4738R1 | TGGTGCGAGTAGTCGATGAG |
| 2633557 | 2634233 | GACG4738F2 | GCTCAATTACACGGTGCTGA |
|  |  | GACG4738R2 | TGAGGTCGTTCTCGTGTGAC |
| 2702173 | 2702852 | GACG4771F | ATATCCTGCACAGCTACTGTCAAT |
|  |  | GACG4771R | TTCCAGTCCATCCACATCAA |
| 2804423 | 2805049 | GACG4797F | TAGGGAAAGAACTGGGCATC |
|  |  | GACG4797R | CATCACTGTGCTTGCACCATA |
| 2873744 | 2874343 | GACG4850F | AGCTCATTCCCACGATGAAG |
|  |  | GACG4850R | CCGGTGTAGTCAACACAGCTT |
| 2976293 | 2976973 | GACG4904F | AGCACCACGTGTTTGTATGTG |
|  |  | GACG4904R | TTGTCCCACCGTTACACTCA |
| 3053607 | 3054264 | GACG4954F1 | CCTGTGAAATAGCCGTCCTC |
|  |  | GACG4954R1 | CCCTCTCAGGCTTAGTGATGA |
| 3105367 | 3106111 | GACG4954F2 | ATGCGAGAGTCGTCATCCTT |
|  |  | GACG4954R2 | CTCCCAGAACTCAGCAAACC |
| 3172433 | 3173065 | GACG4985F | TTCTATTGGCGGCATTTGTT |
|  |  | GACG4985R | AATTCAAGAAGACTGGGCATAAA |
| 3274488 | 3275109 | GACG5034F | TCCTGCAGCTTGGAGTAAGG |
|  |  | GACG5034R | GCCAAGGCTGACCTAGACTG |
| 3349533 | 3350207 | GACG5144F | GGGCCCTTCTCATAAAGCTC |
|  |  | GACG5144R | CCTCCTCCATCTGCTACTGC |
| 3437809 | 3438554 | GACG5222F | CCGTAGCCTACCGTTGTCAT |
|  |  | GACG5222R | GACGAGGGTTTCATCAAGGA |
| 3493027 | 3493734 | GACG5236F | ACTAACAGGGAGAGCGAGCA |
|  |  | GACG5236R | CCACATCTCATTGGCACAAC |
| 3519621 | 3520410 | GACG5350F | TCCCATCATTCATCATCACA |
|  |  | GACG5350R | TCCAGTGTCATCCTGTTTGC |
| 3690908 | 3691510 | GACG5448F | TGGATCATGGTTTGATCGTG |
|  |  | GACG5448R | CTCCTTACGAACCGTTCCAG |
| 3735340 | 3735963 | GACG5480F | ATGGTGACGATGACGAAGGT |
|  |  | GACG5480R | TGCTCGCAAACGTATGTCTC |
| 3823064 | 3823820 | GACG5499F | TAAGCTGCACCAAGGATGTG |
|  |  | GACG5499R | TGATTAGCCTCGCCAAACAT |
| 3905522 | 3906134 | GACG5560F | GCACAGCAGCTCCTTTGTCT |
|  |  | GACG5560R | GTCTGCACCTGCTTGCTCTT |

^*^Positions in LG17 of the reference genomic sequence
