# Supplementary material for: Whole‐genome sequencing reveals small genomic regions of introgression in an introduced crater lake population of threespine stickleback
Source: Ecol Evol. 2016 Mar 2;6(7):2190–204. doi: 10.1002/ece3.2047 (PMC4782248; doi:10.1002/ece3.2047)
Supplement: Supplementary file 11 — Table S2. Number of G. nipponicus‐specific SNPs detectable in the introgression sites in the 19 introgression sites by whole genome sequencing (WGS), simulated RAD sequencing and simulated ddRAD sequencing. [file ECE3-6-2190-s011.docx]

Table S2 Number of *G. nipponicus*-specific SNPs detectable in the introgression sites in the 19 introgression sites by whole genome sequencing (WGS), simulated RAD sequencing and simulated ddRAD sequencing

| LG | Start (bp) | End (bp) | Length (bp) | Number of detectable *G. nipponicus*–specific SNPs | | | |
| --- | --- | --- | --- | --- | --- | --- | --- |
|  |  |  |  | WGS | RAD  SbfI | ddRAD  EcoRI-MspI | ddRAD  SphI-EcoRI |
| 1 | 10,180,001 | 10,528,000 | 348,000 | 904 | 18 | 2 | 0 |
| 2 | 5,324,001 | 5,417,000 | 93,000 | 241 | 2 | 0 | 0 |
| 4 | 31,154,001 | 31,171,000 | 17,000 | 31 | 2 | 1 | 0 |
| 5 | 10,935,001 | 10,949,000 | 14,000 | 11 | 0 | 0 | 0 |
| 6 | 15,682,001 | 15,696,000 | 14,000 | 8 | 0 | 0 | 0 |
| 7 | 5,753,001 | 5,830,000 | 77,000 | 180 | 0 | 0 | 0 |
| 7 | 26,984,001 | 26,995,000 | 11,000 | 13 | 0 | 0 | 0 |
| 8 | 1,662,001 | 1,676,000 | 14,000 | 25 | 0 | 1 | 0 |
| 8 | 18,269,001 | 18,432,000 | 163,000 | 151 | 4 | 4 | 0 |
| 13 | 2,624,001 | 2,642,000 | 18,000 | 43 | 0 | 1 | 1 |
| 13 | 7,717,001 | 7,733,000 | 16,000 | 36 | 0 | 0 | 0 |
| 13 | 19,372,001 | 19,388,000 | 16,000 | 19 | 0 | 0 | 0 |
| 14 | 1,992,001 | 2,003,000 | 11,000 | 25 | 0 | 0 | 0 |
| 14 | 3,320,001 | 3,343,000 | 23,000 | 43 | 3 | 2 | 1 |
| 15 | 11,375,001 | 11,427,000 | 52,000 | 118 | 2 | 1 | 0 |
| 16 | 8,678,001 | 8,691,000 | 13,000 | 22 | 0 | 1 | 0 |
| 17 | 1,169,001 | 1,622,000 | 453,000 | 638 | 32 | 5 | 0 |
| 17 | 4,300,001 | 4,320,000 | 20,000 | 75 | 0 | 0 | 0 |
| 20 | 3,375,001 | 3,406,000 | 31,000 | 61 | 1 | 0 | 0 |

Nineteen introgression sites greater than 10 kb in length found by whole genome sequencing of the Lake Towada population were tested.
